# Dose-dependent immunotoxic mechanisms of celastrol via modulation of the PI3K-Akt signaling pathway

**DOI:** 10.3389/fphar.2025.1567193

**Published:** 2025-05-27

**Authors:** Shaohui Geng, Jingqi Wen, Chunli Shen, Li Liu, Yijin Jiang, Jingyuan Fu, Yiwei Guan, Zi Ye, Yuanhao Wu, Chen Li, Guangrui Huang

**Affiliations:** ^1^ School of Life Science, Beijing University of Chinese Medicine, Beijing, China; ^2^ School of Traditional Chinese Medicine, Beijing University of Chinese Medicine, Beijing, China; ^3^ School of Acupuncture, Moxibustion and Tuina, Beijing University of Chinese Medicine, Beijing, China; ^4^ School of Chinese Pharmacy, Beijing University of Chinese Medicine, Beijing, China; ^5^ The First Affiliated Hospital of Tianjin University of Traditional Chinese Medicine, Tianjin, China; ^6^ Department of Dermatology, Tianjin Institute of Integrative Dermatology, Tianjin Academy of Traditional Chinese Medicine Affiliated Hospital, Tianjin, China

**Keywords:** celastrol, immunotoxicity, PI3K-Akt signaling pathway, network toxicology, molecular docking

## Abstract

**Ethnopharmacological relevance:**

Celastrol, a bioactive compound from *Tripterygium wilfordii Hook.f.*, is known for its anti-inflammatory and immunomodulatory effects, but its immunotoxicity is underexplored. This study investigates the mechanisms of celastrol-induced immunotoxicity, focusing on the PI3K-Akt signaling pathway, a key regulator of immune function.

**Materials and methods:**

An integrative approach combining network toxicology, molecular docking, and experimental biology identified molecular targets in celastrol-induced immune dysfunction. Network toxicology mapped key pathways, and molecular docking predicted interactions with immune-related proteins. High-dose celastrol (10 mg/kg) was administered to C57BL/6J mice, followed by a histopathological analysis of the thymus and spleen. RNA-Seq evaluated gene expression in immune pathways, and IHC/mIHC validated PI3K-Akt signaling pathway protein expression.

**Results:**

Network toxicology identified the PI3K-Akt signaling pathway as a key target of celastrol’s immunotoxic effects. High-dose celastrol caused histopathological damage in the thymus and spleen, including lymphocyte depletion and immune cell infiltration. RNA-Seq showed upregulation of critical genes in the PI3K-Akt signaling pathway (Egfr, Pik3c, Akt3), linked to cell proliferation and survival. IHC confirmed increased expression of EGFR, AKT, PIK3, and mTOR, with decreased PTEN. mIHC revealed elevated macrophage activation and inflammation. In contrast, low-dose celastrol suppressed PI3K-Akt signaling by downregulating mTOR, indicating dose-dependent modulation of immune function.

**Conclusion:**

Our study demonstrates the dose-dependent immunotoxic effects of celastrol, which is toxic in high doses caused by activation of the PI3K-Akt signaling pathway, while low doses offer protection by blocking this signaling pathway. These findings emphasize the importance of dose selection in therapeutic and safety contexts, enhancing understanding of celastrol’s biological effects and its clinical potential in immune-related diseases.

## Graphical Abstract

**Figure:**
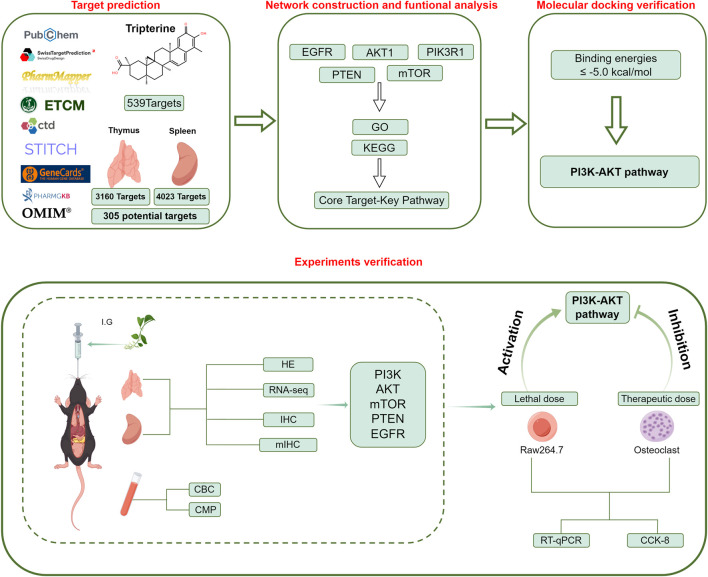
The dose-dependent immunotoxic mechanisms of celastrol are caused by modulating the PI3K-Akt signaling pathway.

## 1 Introduction

Celastrol, a bioactive triterpenoid lactone epoxide derived from the root bark of *Tripterygium wilfordii Hook.f.* (Chen et al., 2018), is a key component of traditional Chinese medicine, as documented in the *Shennong’s Herbal Classic*. Modern pharmacological studies have demonstrated that celastrol possesses anti-inflammatory, anti-tumor, and neuroprotective pharmacological activities across a broad spectrum (Hou et al., 2020; Li et al., 2024; Liu et al., 2022; Tan et al., 2025; Zhu et al., 2024). Notably, celastrol exhibits therapeutic potential in various pathological conditions, including obesity (Shi et al., 2020), cancer (Wang et al., 2023a; Wu et al., 2024), and autoimmune disease (Deng et al., 2021; Pu and Ye, 2025; Wang, D.D. et al., 2024; Zeng et al., 2024).

Although celastrol has shown promising pharmacological effects as a therapeutic agent, its unique toxic side effects cannot be ignored. In human rheumatoid synovial fibroblasts, celastrol exhibited dose-dependent cytotoxicity, with concentrations of 1, 2, 5, and 10 μM significantly inhibiting cell proliferation, inducing DNA damage, cell cycle arrest, and apoptosis (Gan et al., 2015). In an LPS-induced septic mouse model, celastrol aggravated liver damage by promoting morphological changes and oxidative stress (Wu et al., 2018). Liu et al. (2024) found that celastrol induces nephrotoxicity through dysfunction in ATP metabolism and mitochondria-dominated apoptosis. Furthermore, Shirai et al. (2023) demonstrated that celastrol inhibits humoral immune responses and autoimmune diseases by targeting the COMMD3/8 complex. Moreover, Wen et al. (2023) showed that celastrol induces premature ovarian insufficiency by inducing apoptosis in granulosa cells. Despite these insights into celastrol’stoxicological profiles, the mechanisms underlying its immunotoxicity remain poorly understood, and further research is needed to elucidate its potential targets and pharmacological mechanisms.

The thymus, as a central immune organ, governs T cell differentiation and maturation, while the spleen, as a peripheral immune organ, regulates the activation of adaptive immune responses. These two organs integrate into a dynamic immune regulatory network through T cell migration and functional synergy (Cesta, 2006; Miller, 1961). Studies have shown that immunotoxic agents, such as Newcastle disease virus (Wang et al., 2024) and T-2 toxin (Chen et al., 2025), induce thymic lymphocyte depletion and splenic tissue necrosis in animals, potentially via NLRP3 inflammasome activation, apoptosis, and oxidative stress-mediated mitochondrial apoptotic pathways. However, existing studies primarily focus on the toxicity of individual organs, leaving the systemic immunosuppressive mechanisms of celastrol largely unexplored.

Dysregulated activation of the PI3K-Akt signaling pathway has been implicated in various acute toxic responses. High-dose flufenacet triggers apoptosis and cell cycle arrest by modulating the PI3K-Akt signaling pathway, leading to developmental toxicity in zebrafish (An et al., 2023). Mercuric chloride significantly upregulates PI3K, AKT, and STAT-5A expression, contributing to acute nephrotoxicity in rats (Kadry and Megeed, 2022). Bisphenol A disrupts DPY30 expression, inducing PI3K-Akt pathway dysregulation and cell cycle arrest, ultimately causing testicular toxicity in mice (He et al., 2022). These findings suggest that dysregulation of the PI3K-Akt signaling pathway may contribute to acute multi-organ toxicity by impairing cell cycle progression and apoptotic homeostasis.

This study aims to systematically investigate the dose-dependent immunotoxicity of celastrol mediated via the PI3K-Akt signaling pathway. Using network toxicology and molecular docking, we predict potential immunotoxic targets and validate these findings through *in vivo* assays and osteoclast differentiation tests. This study will clarify the molecular mechanisms of celastrol immunotoxicity through the PI3K-Akt signaling pathway, providing a scientific basis for its safe application, optimized therapeutic strategies, and clinical translation.

## 2 Materials and methods

### 2.1 Materials and instruments

**FIGURE 1:**
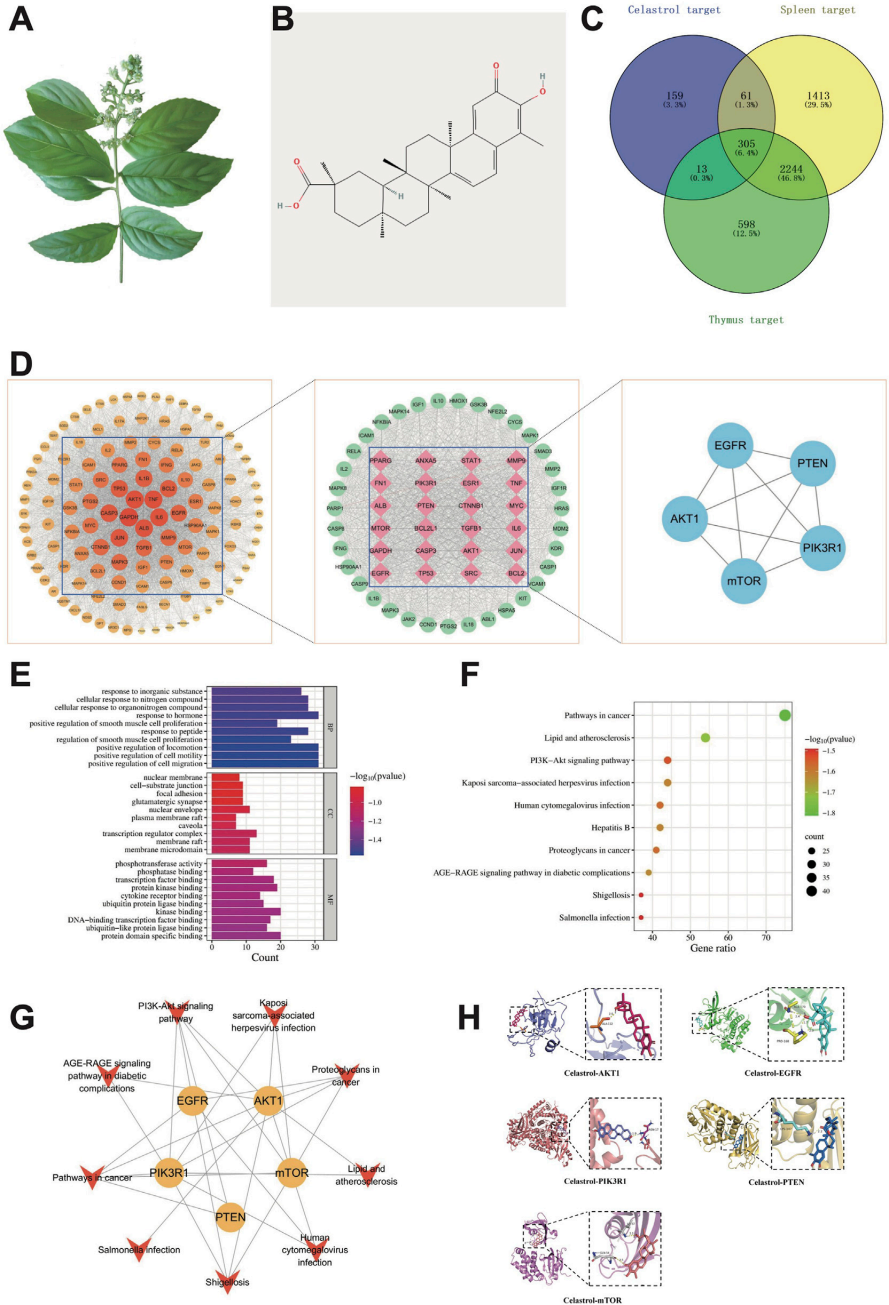
Network toxicology and molecular docking analysis of celastrol-induced immunotoxicity. **(A)** Tripterygium wilfordii herb; **(B)** Two-dimensional structural diagram of celastrol; **(C)** Common targets of immunotoxicity induced by celastrol; **(D)** Flowchart for core targets identification; **(E)** GO function enrichment analysis; **(F)** KEGG pathway enrichment analysis; **(G)** “Target-pathway” network; **(H)** Molecular docking of celastrol with core targets.

Celastrol, from Tripterygium wilfordii Hook.f. (Figures 1A,B, lot number N01GB166246), purity ≥98%, sourced from Shanghai Yuanye Bio-Technology Co., Ltd. XN-1000V Automated Hematology Analyzer (Sysmex Corporation, Germany); AU680 Automated Biochemistry Analyzer (Beckman Coulter, China); Centrifuge; Leica Aperio Versa Super-Resolution Microscopy System for Tissue Imaging (Leica Microsystems, USA).

### 2.2 Network toxicology analysis

#### 2.2.1 Databases and tools

For the network toxicology study, we utilized various databases and tools including PharmMapper (http://www.lilab-ecust.cn/pharmmapper), STITCH (http://stitch.embl.de/), ETCM (http://www.tcmip.cn/ETCM/index.php/Home/), CTD (http://ctdbase.org/, corrected link), PubChem (https://pubchem.org/), UniProt (http://www.uniprot.org/), OMIM (http://www.omim.org/), GeneCards (https://www.genecards.org/), STRING (https://string-db.org/), Metascape (https://metascape.org/), Swiss Target Prediction (http://www.swisstargetprediction.ch/), Venn diagram platform (http://bioinfogp.cnb.csic.es/tools/venny/index.html). Cytoscape 3.10.2 for visualization, Graphpad prism10.0 and SPSS 27.0 for data analysis.

#### 2.2.2 Prediction of celastrol’s targets

Celastrol’s two-dimensional structure in SDF format and Canonical SMILES sequence were obtained from the PubChem database for network toxicology analysis. Potential targets of celastrol were identified through Swiss Target Prediction, PharmMapper, ETCM, CTD, and STITCH databases. Target gene names were standardized using the UniProt database, restricting the species to *Homo sapiens* for toxic target identification.

#### 2.2.3 Prediction of immunotoxicity targets

Keywords associated with immune system diseases, such as “Thymic toxicity,” “Thymus injury,” “Spleen toxicity,” and “Spleen injury”, were utilized to query GeneCards, PharmGKB, and OMIM databases. In GeneCards, a higher Score value indicates a closer association between a target and the disease. Targets exceeding the median score were empirically selected as potential toxic targets (Yang et al., 2022; Zhao et al., 2023). After merging targets from the three disease databases and removing duplicates, toxic targets for the thymus and spleen were obtained. Celastrol-related targets and toxic targets of the thymus and spleen were analyzed using a Venn diagram to identify common targets.

#### 2.2.4 Construction of the target interaction network

The common targets of celastrol-induced immunotoxicity were imported into the STRING database. A protein-protein interaction (PPI) network was generated by selecting Multiple Proteins and *H. sapiens*, setting the confidence threshold to 0.400, and hiding discrete nodes. The PPI network data were then analyzed in Cytoscape 3.10.2where the “Network Analyzer” function was used for topological analysis. Key targets were determined using degree centrality (DC), betweenness centrality (BC). PPI relationships and clustering analysis were conducted to identify key protein interactions and their functional relevance to celastrol-induced immunotoxicity.

#### 2.2.5 GO biological analysis and KEGG pathway enrichment analysis

The intersection targets of celastrol-induced thymic and splenic toxicity were imported into the Metascape database for Gene Ontology (GO) biological analysis (Bindea et al., 2009) and Kyoto Encyclopedia of Genes and Genomes (KEGG) pathway enrichment analysis (Kanehisa et al., 2008), with a significance threshold of *P* < 0.01. The GO analysis results, including Biological Process (BP), Cellular Component (CC), and Molecular Function (MF), as well as KEGG enrichment results, were saved in TSV format. After filtering, the results were visualized using a bioinformatics platform.

### 2.3 Molecular docking validation

Molecular docking was conducted using AutoDock software to evaluate interactions between celastrol and the key targets identified in Section 2.2.4. The SDF file of the 3D structure of celastrol was retrieved from the PubChem database and converted into a PDB file using OpenBabel software. Target protein structures in PDB format were obtained from the Protein Data Bank (PDB, https://www.rcsb.org/) based on protein names. AutoDock Tools 1.5.6 was used to add hydrogen atoms, designate ligands, and save files in PDBQT format. The processed protein structures were also converted into PDBQT format after removing water molecules and adding hydrogen atoms. Molecular docking simulations were then executed, and binding energy values were calculated. Docking results were visualized using Pymol software (Yuan et al., 2017).

### 2.4 Animal experiments

#### 2.4.1 Animals

Twenty SPF-grade male C57BL/6J mice, with a body weight of (25 ± 5) g, were purchased from Beijing Vital River Laboratory Animal Technology Co., Ltd. The mice were housed in the Experimental Animal Center of Beijing University of Chinese Medicine under standard conditions, including a 12-h light/12-h dark cycle.

#### 2.4.2 Acute toxicity study

The animals were acclimatized for 2 weeks and then randomly assigned to either the control or celastrol group. Based on the equivalent dose ratio of 0.0026 between humans and mice, the calculated therapeutic dose of celastrol was 1.1 mg/kg/day. According to preliminary experimental data, a dose of 10 mg/kg (10 times the therapeutic equivalent dose) was selected for the celastrol group. Mice were administered daily for 7 days with a dosing volume of 10 mL/kg every 24 h. Distilled water served as the control, and an equivalent volume was administered to the control group based on body weight. After the final administration, mice were fasted and water-deprived for 12 h before blood and organ collection for biochemical and histological analysis. This study was approved by the Animal Ethics Committee of Beijing University of Chinese Medicine (BUCM-2024030106-1208).

#### 2.4.3 Behavioral observation, survival rate, and body weight analysis

Daily observations of mouse coat color, feeding and water intake, activity levels, and vital signs were conducted during the drug administration period to monitor for signs of toxicity. Additionally, body weight was recorded daily from the first day of drug administration.

#### 2.4.4 Blood routine test

Blood samples were collected via enucleation and transferred into EDTA-coated tubes for hematological analysis. An automated hematology analyzer was performed to obtain cell classification and counts, including white blood cells (WBC), neutrophils (NEUT), and lymphocytes (LY).

#### 2.4.5 Serum biochemical indices analysis

Blood samples were allowed to clot at room temperature for 2 h, followed by centrifugation at 4°C for 20 min at 3,000 rpm to collect the serum. Alanine aminotransferase (ALT), alkaline phosphatase (ALP), and total bilirubin (TBIL) levels in the serum were measured using an automated biochemical analyzer to assess liver function. Blood urea nitrogen (BUN) and serum creatinine (SCR) levels were also detected to evaluate the kidney function.

#### 2.4.6 Organ index and histological examination

Thymuses and spleens were harvested from each group, with fat tissue and fascia removed before weighing and photographing. The organ index was calculated as the organ weight (mg) per body weight (g). The organs were fixed in 4% paraformaldehyde for subsequent pathological examination. After dehydration, paraffin embedding, sectioning, dewaxing, hematoxylin-eosin (HE) staining, clearing, and mounting, histological changes in the thymuses and spleens were observed and photographed under a 400× optical microscope.

### 2.5 Osteoclast induction and differentiation experiment

RAW264.7 cells were seeded in α-MEM supplemented with 10% FBS and incubated for 12 h. Subsequently, 150 ng/mL RANKL was added to induce osteoclast differentiation, with the medium refreshed every 3 days while maintaining RANKL supplementation. Multinucleated osteoclasts became detectable after 4 days of differentiation, with a substantial increase observed by days 5–6.

### 2.6 Tartrate-resistant acid phosphatase (TRAP) staining

Cells were washed three times with PBS, followed by fixation in pre-cooled TRAP fixative at 2°C–8°C for 30 s. After washing with distilled water, TRAP incubation solution was added to cover the cells and incubated at 37°C for 45–60 min. Hematoxylin staining was performed for 2 min, followed by a 10-min wash in tap water for nuclear counterstaining. TRAP-positive osteoclasts were identified by purplish-red cytoplasm and blue-stained nuclei under a microscope.

### 2.7 RNA-seq analysis

On the seventh day, euthanize the mice and harvest their thymuses and spleens, which are then promptly placed into liquid nitrogen. Total RNA of the tissue samples were extracted andfollowed by the assessment of theirconcentration and purity by using a Nanodrop 2000. Agarose gel electrophoresis was used to verify RNA integrity, while the Agilent 2100 system determined the RNA integrity number (RIN) values. The preparation of sequencing libraries for each RNA sample was carried out using the Ion total RNA-Seq Kit v2 (Life Technologies, USA), adhering to the manufacturer’s guidelines. Subsequently, the cDNA libraries underwent sequencing on an Illumina NovaSeq 6000 platform, adhering to standard protocols.

Statistical analysis of transcriptome sequencing data was performed using the Meiji Cloud Platform (https://cloud.majorbio.com/). Differential gene expression analysis was conducted using DESeq2, with statistical significance set at *P* < 0.05. GO enrichment analysis was performed using Goatools (Klopfenstein et al., 2018) (https://github.com/tanghaibao/Goatools), considering *P* < 0.05 as the threshold for significant functional enrichment. KEGG pathway enrichment analysis was conducted using custom R scripts (Varet et al., 2016) with the same significance threshold. Gene Set Enrichment Analysis (GSEA) was performed to identify significantly enriched pathways, ranking gene sets based on differential expression. The enrichment score (ES) was calculated for each predefined gene set, and statistical significance was determined using a permutation-based method, with a false discovery rate (FDR) < 0.05 considered as significant pathway enrichment.

### 2.8 Immunohistochemical (IHC)

Based on the prediction results of the previous network toxicology analysis regarding the immunotoxicity of celastrol, relevant predicted targets and major injury mechanisms were validated. Immunohistochemistry was utilized to investigate the effects of celastrol on the expression of EGFR, PI3K, AKT, mTOR and PTEN in the thymuses and spleens.

### 2.9 Multiplex immunohistochemical (mIHC)

To evaluate the toxic effects of celastrol on inflammation and macrophage polarization, mIHC was performed using the following markers: Anti-IL-1β (Abcam, ab283818), TNF-α Monoclonal Antibody (Proteintech, 60291-1-Ig), Anti-iNOS (Abcam, ab210823), and Anti-Mannose Receptor (Abcam, ab64693). The sections were then placed in a humidified chamber and incubated overnight at 4°C or for 30 min at 37°C. After incubation, a properly diluted secondary antibody was applied dropwise to the slides, ensuring thorough coverage of the tissue. DAPI staining solution was then added dropwise and incubated for 5 min at room temperature. Finally, the slides were sealed using an anti-fluorescence quencher.

### 2.10 CCK-8 assay

RAW264.7 cells were seeded in 96-well culture plates at a density of 1 × 10^4^ cells/well and incubated overnight at 37°C in a humidified incubator with 5% CO_2_. The next day, cells were washed twice with PBS, and 100 μL of fresh medium was added. After 24 h, celastrol was administered at final concentrations of 100, 50, 10, 5, 1, 0.5, and 0.1 μmol/L. After 48 h of treatment, 90 μL of fresh medium and 10 μL of CCK-8 solution were added to each well, followed by incubation at 37°C for 2 h. The optical density (OD) at 450 nm was measured using a microplate reader to assess cell viability.

### 2.11 Real-time quantitative polymerase chain reaction (RT-qPCR)

RAW264.7 cells were seeded in α-MEM containing 10% FBS and incubated for 12 h. RANKL (150 ng/mL) was then added, and the medium was refreshed every 3 days with RANKL supplementation. TRAP staining was performed to confirm osteoclast differentiation, with successfully induced osteoclasts identified by their purplish-red cytoplasm and blue-stained nuclei.

Total RNA was extracted using the Cell Fast RNA Extraction Kit for Animal (ABclonal, RK30120) according to the manufacturer’s instructions.cDNA was synthesized using the ABScript Neo RT Master Mix for qPCR (ABclonal, RK20432). Real-time PCR was performed using SYBR Green (ABclonal, RK21203) on a CFX96 Real-Time PCR Detection System (Bio-Rad). Relative gene expression levels were calculated using the 2 (-△△Ct) method, with β-actin as the internal control. Primer sequences are provided in Table 1.

**TABLE 1:** Primers for RT-qPCR analysis of gene transcript expression.

Gene	Forward primer	Reverse primer
AKT	TAT​TGA​GCG​CAC​CCT​TCC​AT	GAC​CTG​TGG​CCT​TCT​CCT​TC
PTEN	TTG​AAG​ACC​ATA​ACC​CAC​CAC​A	ATC​ATT​ACA​CCA​GTC​CGT​CCC​T
mTOR	ACC​GGC​ACA​CAT​TTG​AAG​AAG	CTC​GTT​GAG​GAT​CAG​CAA​GG
EGFR	CAA​TGT​TCC​CAT​CGC​TGT​CGT	TGT​CTT​TGC​ATG​TGG​CCT​CAT
PI3K	ACA​CCA​CGG​TTT​GGA​CTA​TGG	GGC​TAC​AGT​AGT​GGG​CTT​GG
β-actin	TTC​TAC​AAT​GAG​CTG​CGT​GTG	AGA​GGC​GTA​CAG​GGA​TAG​CA

### 2.12 Statistical analysis

All quantitative data were analyzed using SPSS 27.0 statistical software. Data were expressed as mean ± standard deviation (SD). Independent sample *t*-tests were used for pairwise comparisons, with a significance level set at α = 0.05.

## 3 Results

### 3.1 Network toxicology analysis

#### 3.1.1 Target prediction

Potential targets of celastrol were systematically identified through multi-database integration: 104 targets were initially screened from the Swiss Target Prediction database (Probability >0) (Daina et al., 2019); 282 targets were retrieved from PharmMapper using high-confidence thresholds (Fit Score ≥5.0, *P* < 0.05) (Wang et al., 2017); 10 targets with reliable interactions (Combined Score ≥0.7) were selected from STITCH(Szklarczyk et al., 2016); 14 targets were extracted from, ETCM(Xu et al., 2019) (P < 0.05) and 210 literature-supported targets (Interactions ≥1) were curated from CTD (Davis et al., 2023). After eliminating duplicate entries, 539 distinct toxicological targets for celastrol were obtained. To further investigate the association with immune-related toxicity, a comprehensive search was conducted across the GeneCards, PharmGKB, DISGENET, and OMIM databases, which led to the identification of 3,160 thymus toxicity-related targets and 4,023 spleen toxicity-related targets. Following data cleaning to remove redundancies, a Venn diagram analysis was performed to integrate the targets of celastrol with those related to thymus and spleen toxicity. This process identified a total of 305 potential targets associated with celastrol-induced immunotoxicity, as illustrated in Figure 1C. In addition, the Venn diagram and specific targets of the celastrol-spleen-thymus interaction are presented in Supplementary Figure S11 and Supplementary Table S2, respectively.

#### 3.1.2 Target interaction network analysis

The immunotoxicity-related potential targets of celastrol were input into the STRING database (Szklarczyk et al., 2019) to explore protein-protein interaction (PPI) relationships. These interactions were subsequently analyzed using Cytoscape software for topological parameter calculations. Core targets were selected through a two-step screening process, with the first screening based on the median values of various parameters, where operational values exceeding the median of three parameters were considered. After the first round of screening, 124 targets were retained, and the corresponding PPI network is shown in Figure 1D. In this network, the node size and color intensity are proportional to the Degree value, which reflects the significance of each target in the network, with higher Degree values indicating a more critical role. Following the second screening, 59 core targets were identified, as depicted in Figure 1D. The resulting network consists of 59 nodes and 1,558 edges, with an average Degree value of 52.814. Key target information from the PPI network is summarized in Supplementary Table S1. Based on the topological analysis, the top five core targets with the highest combined centrality rankings (centrality, closeness centrality, and betweenness centrality) were identified as EGFR, AKT1, PIK3R1, PTEN, and mTOR, as illustrated in Figure 1D and Table 2. Further details on the PPI relationships and clustering analysis can be found in Supplementary Figures S1, S2.

**TABLE 2:** Core targets information.

Target symbol	Target name	UniProt ID
EGFR	Epidermal growth factor receptor	P00533
PTEN	Phosphatidylinositol 3,4,5-trisphosphate 3-phosphatase and dual-specificity protein phosphatase PTEN	P60484
PIK3R1	Phosphatidylinositol 3-kinase regulatory subunit alpha	P27986
AKT1	RAC-alpha serine/threonine-protein kinase	P31749
mTOR	Serine/threonine-protein kinase mTOR	P42345

#### 3.1.3 GO and KEGG pathway analysis

GO biological process (BP) analysis of the 59 core targets identified a total of 1,463 enriched terms, primarily associated with the positive regulation of cell migration, cell motility, and smooth muscle cell proliferation. Cellular component (CC) analysis yielded 62 enriched terms, mainly related to membrane rafts, membrane microdomains, and transcription regulator complexes. Additionally, molecular function (MF) analysis identified 95 enriched terms, with a primary focus on protein domain-specific binding, ubiquitin-like protein ligase binding, and kinase binding.

KEGG pathway analysis revealed enrichment in 176 signaling pathways, with the most significant pathways including Pathways in cancer, Lipid and atherosclerosis, and the PI3K-Akt signaling pathway. Notably, 44 targets, including EGFR, AKT1, PIK3R1, PTEN, and mTOR, were mapped to the PI3K-Akt signaling pathway, highlighting its potential association with celastrol-induced immunotoxicity. Using a significance threshold of *P* < 0.01, the top 10 GO-enriched terms and KEGG pathways were selected for visualization, as depicted in Figures 1E,F. In these figures, the Y-axis represents the pathway names, while the X-axis denotes the proportion of genes enriched in each pathway. The bubble size corresponds to the number of enriched genes, and the color gradient indicates the statistical significance of enrichment.

#### 3.1.4 Core target-pathway analysis

To further elucidate the molecular mechanisms underlying celastrol-induced immunotoxicity, a “core target-pathway” interaction network was constructed, as illustrated in Figure 1G. In this network, yellow diamond-shaped nodes represent the core targets, while red arrows signify the key signaling pathways. The analysis revealed that all five core targets—EGFR, AKT1, PIK3R1, PTEN, and mTOR—were significantly enriched in the top-ranked KEGG pathways. Among these, the PI3K-Akt signaling pathway exhibited the highest number of core targets, suggesting its pivotal role in mediating celastrol’s immunotoxic effects. This network analysis provides critical insights into the mechanistic basis of celastrol-induced immunotoxicity and underscores the importance of the PI3K-Akt signaling pathway in regulating these effects.

### 3.2 Molecular docking

Molecular docking simulations were conducted using AutoDock software to predict the binding interactions between celastrol and its core targets, including EGFR, PTEN, PIK3R1, AKT1, and mTOR. The binding energy values, as summarized in Table 3, were calculated as −6.89, −7.22, −5.92, −8.11, and −5.63 kcal/mol. Notably, all binding energies were ≤−5.0 kcal/mol, which is considered indicative of strong binding affinity (Ferreira et al., 2015). These results suggest a high likelihood of celastrol interacting with the identified core targets, supporting its potential role in modulating these pathways. Finally, the docking interactions were visualized using Pymol software, as shown in Figure 1H, providing a structural representation of celastrol binding to its target proteins.

**TABLE 3:** Binding energy between celastrol and core targets.

Target	PDB ID	Binding energy (kcal/mol)
EGFR	7KP6	−6.89
PTEN	5BZZ	−7.22
PIK3R1	8BCY	−5.92
AKT1	8R5K	−8.11
mTOR	6M4U	−5.63

### 3.3 Celastrol acute toxicity study in mice

#### 3.3.1 Behavioral observations in mice

**FIGURE 2:**
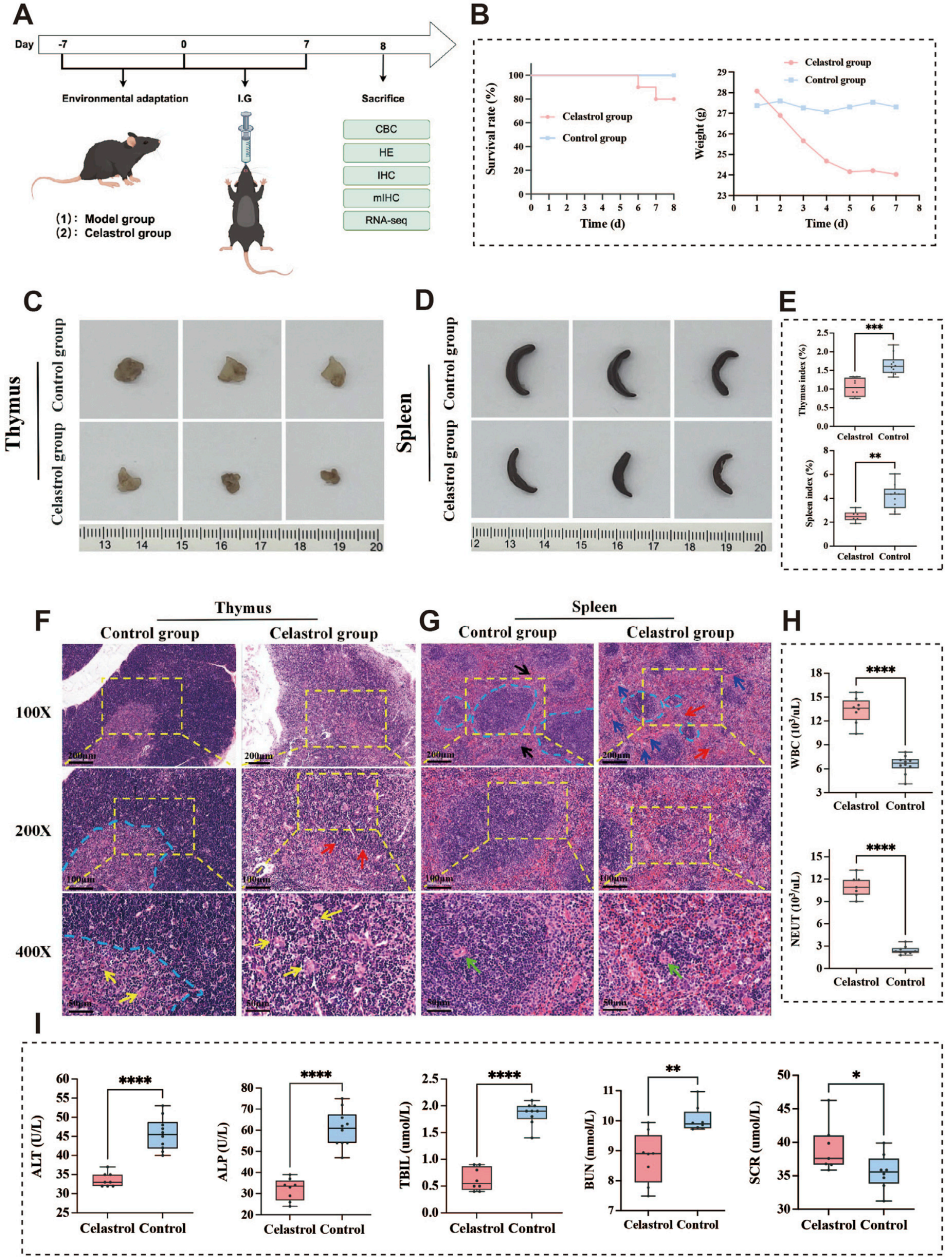
Analysis of acute toxicity animal experiment results of celastrol. **(A)** The acute toxicity animal experiment design; **(B)** The results of survival curve and body weight changes; **(C,D)**. Morphological observation of thymus and spleen; **(E)** Comparison of thymus and spleen index; **(F,G)**. Histopathological examination of thymus and spleen tissue sections in mice; **(H)** The results of blood routine examination **(I)** The results of Serum biochemical tests. (mean ± SD, n = 3, ^*^ *P* < 0.05, ^**^ *P* < 0.01, ^***^ *P* < 0.001, ^****^ *P* < 0.0001).

The experimental design is illustrated in Figure 2A. Mice in the control group maintained normal feeding behavior, exhibited active movements, glossy fur, and quick responses throughout the study. Notably, no mortality occurred in this group until day 7. In contrast, the celastrol-treated mice showed pronounced behavioral changes starting from day 2 of administration. These mice displayed signs of lethargy, dull and dry fur, and a decrease in spontaneous activity. Their responses became slower, and by day 3, they developed anorexia, loose stools, and dried feces adhered to the perianal and tail base areas. These behavioral alterations highlight the significant adverse effects of celastrol on the overall health of the mice.

#### 3.3.2 Survival curve and body weight analysis

The survival rate and body weight changes over the 7-day period are shown in Figure 2B. Notably, 1 mouse from the celastrol group died on both the sixth and seventh days of administration. The body weight of the control group mice remained stable, with only slight fluctuations. However, the mice in the celastrol group experienced a substantial weight loss, especially noticeable by day 7. Daily body weight measurements were recorded, and the average weight for each group was calculated. Statistical analysis revealed a significant difference in body weight changes between the celastrol and control groups (*P* < 0.001), indicating that celastrol administration has a detrimental effect on body weight and survival. Inter-group differences were analyzed by *t*-test based on the average body weight data collected over the 7 days, revealing statistical significance between the two groups.

#### 3.3.3 Organ index comparison of thymus and spleen

Morphological observations revealed that the thymus and spleen in the celastrol-treated mice were notably smaller than those in the control group, as shown in Figures 2C,D. Organ index calculations demonstrated a significant decrease in the thymus index (*P* < 0.001) and the spleen index (*P* < 0.01) in the celastrol group compared to the control group. These findings, presented in Figure 2E, suggest that celastrol administration may suppress the function of immune-related organs, including the thymus and spleen.

#### 3.3.4 Histopathology of thymus and spleen

Histopathological examination following HE staining revealed the microscopic characteristics of thymus tissue, as shown in Figure 2F. In the control group, the thymus exhibited a normal microstructure, with a distinct boundary between the darker-staining cortex and the lighter-staining medulla, indicated by the blue dashed line. The cortex and medulla contained densely packed lymphocytes, with scattered thymic corpuscles of varying sizes (yellow arrows) in the medulla. In contrast, thymus sections from celastrol-treated mice exhibited significant structural damage, characterized by a loose structure and indistinct boundaries between the cortex and medulla (red arrows), along with numerous vacuoles and spaces. The cortex was thinned with pale staining and a marked reduction in thymocyte numbers, while the medulla showed sparse lymphocyte arrangement and an increase in thymic corpuscles (yellow arrows).

Microscopic analysis of spleen tissue is shown in Figure 2G. The spleen in the control group displayed clear structural organization, with distinct borders between the red and white pulp (blue dashed line). The white pulp contained numerous splenic nodules and periarterial lymphatic sheaths around the splenic artery (black arrows), which were of moderate size and thickness, with neatly arranged lymphocytes. In comparison, the spleen in the celastrol group exhibited noticeable pathological changes, including blurred boundaries between the red and white pulp (red arrows) and a looser cell arrangement. The red pulp showed an increased number of splenic cords (blue arrows) and higher blood cell density in the splenic sinusoids. The white pulp was reduced in size (blue dashed line), with smaller splenic nodules and a decreased number and thickness of periarterial lymphatic sheaths around the splenic artery.

#### 3.3.5 Blood routine and biochemical tests

The impact of celastrol administration on WBC and NEUT counts in mouse blood is shown in Figure 2H. The effect of celastrol on LY count is shown in Supplementary Figure S3 (Supplementary Material). Compared to the control group, the celastrol group exhibited significantly elevated WBC and NEUT counts (*P* < 0.0001), suggesting the induction of an inflammatory response. These findings indicate that celastrol treatment may trigger an inflammatory reaction and an immunosuppressive effect.

The effects of celastrol on serum ALP, ALT, TBIL, BUN, and SCR levels are presented in Figure 2I. The effect of celastrol on serum AST levels is shown in Supplementary Figure S4. Compared to the control group, the celastrol group exhibited significantly reduced serum levels of ALP, ALT, and TBIL (*P* < 0.0001), indicating substantial liver damage caused by high doses of celastrol. This damage may be mediated through the activation of inflammatory pathways in the liver, leading to hepatocyte death and hepatic dysfunction. Additionally, serum BUN levels were significantly decreased (*P* < 0.01), while SCR levels were significantly increased (*P* < 0.05) in the celastrol group, suggesting potential renal injury or a reduced capacity for renal clearance.

### 3.4 RNA-seq analysis of thymus

The clean data from all thymus tissue samples exceeded 6.05 Gb, with the Q30 base percentage greater than 96.55%, indicating high-quality data. The clean reads from each sample were aligned to the specified reference genome, achieving alignment rates between 97.88% and 98.41%. The results of thymus transcriptome sequencing quality control are presented in Supplementary Figures S10A, C, E.

**FIGURE 3:**
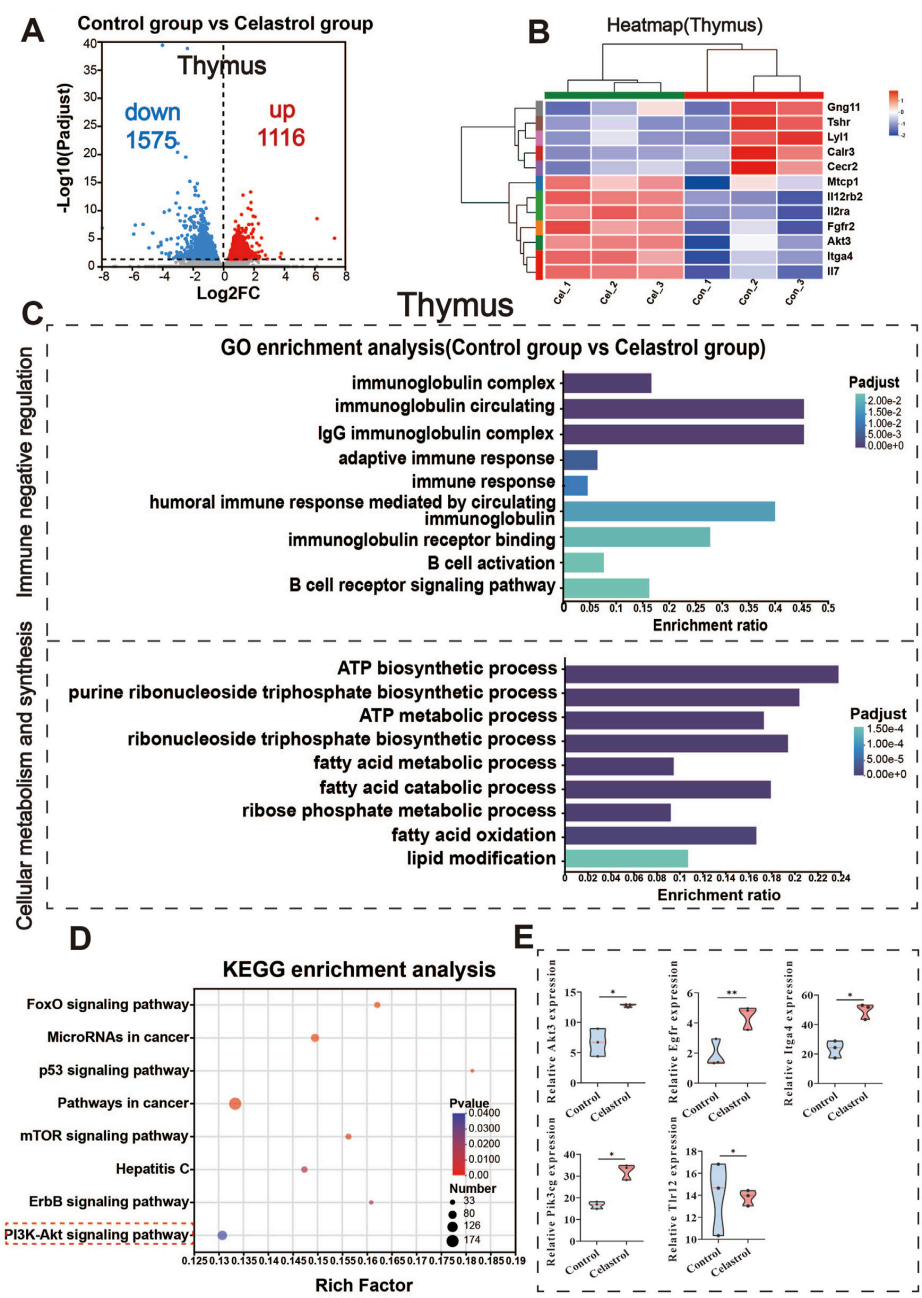
RNA-Seq analysis of thymus between celastrol and control group. **(A)** Volcanic map of differentially expressed genes in thymus; **(B)** Clustering heatmap of differentially expressed genes in thymus; **(C)** GO functional enrichment analysis of differentially expressed genes in thymus; **(D)** KEGG pathway enrichment analysis of differentially expressed genes in thymus; **(E)** Differential expression of key genes in the PI3K-Akt signaling pathway in thymus. (mean ± SD, n = 3, ^*^ *P* < 0.05, ^**^ *P* < 0.01, ^***^ *P* < 0.001).

Differential gene expression analysis was performed using DESeq2, with the Benjamini–Hochberg (BH) method for multiple testing correction. The criteria for identifying differentially expressed genes (DEGs) were |log2FC| ≥ 1 and padjust <0.05. Compared to the control group, 1,116 genes were upregulated in the thymus tissue of the celastrol group, including members of the serine/threonine protein kinase B (AKT) family, such as Akt3. In addition, 1,575 genes were downregulated, including members of the Toll-like receptor (TLR) family, such as Tlr12. These results are presented in Figures 3A,B.

The results of the Gene Ontology (GO) functional enrichment analysis are shown in Figure 3C. Differentially expressed mRNAs in the thymus of the celastrol group were significantly enriched in 162 cellular components, 64 molecular functions, and 458 biological processes. Based on the immune-related functional roles and mechanisms, these differentially expressed genes were categorized into two main groups: immune negative regulation and cell metabolism and synthesis. Immune negative regulation included processes such as adaptive immune response and B cell activation, both of which are negatively regulated. The cell metabolism and synthesis group involved changes in processes like ribose phosphate metabolic process and ATP biosynthesis. Additional Gene Set Enrichment Analysis (GSEA) results for other signaling pathways are provided in the Supplementary Material (Supplementary Figures S8A–D).

The results of the KEGG pathway enrichment analysis are displayed in Figure 3D. The differentially expressed mRNAs in the thymus of the celastrol group were significantly enriched in 113 pathways (*P* < 0.05), including the PI3K-Akt signaling pathway, mTOR signaling pathway, and p53 signaling pathway. In the transcriptomic analysis of thymus tissue, significant upregulation was observed in core genes within the PI3K-Akt pathway considerable, such as Egfr, Pik3c, Akt3, and Igta4, while Tlr12 was notably downregulated, as shown in Figure 3E. The upregulation of Egfr, Pik3c, and Akt3, key regulatory genes in the PI3K-Akt pathway, is involved in regulating cell proliferation and survival (Yu and Cui, 2016), while the downregulation of Tlr12 may further disturb immune response balance (Fitzgerald and Kagan, 2020; Kawai and Akira, 2011). These findings align with the predictions from earlier network toxicology analysis, suggesting that the PI3K-Akt signaling pathway plays a pivotal role in celastrol-induced immunotoxicity. The differential gene expression of selected genes in the PI3K-Akt signaling pathway within the thymus is shown in Supplementary Figure S5.

### 3.5 RNA-seq analysis of spleen

The clean data from all spleen tissue samples exceeded 5.94 Gb, with a Q30 base percentage greater than 96.15%, indicating high data quality. The clean reads from each sample were aligned with the specified reference genome, achieving alignment rates between 97.78% and 98.02%. The results of spleen transcriptome sequencing quality control are presented in Supplementary Figures S10B, D, F.

**FIGURE 4:**
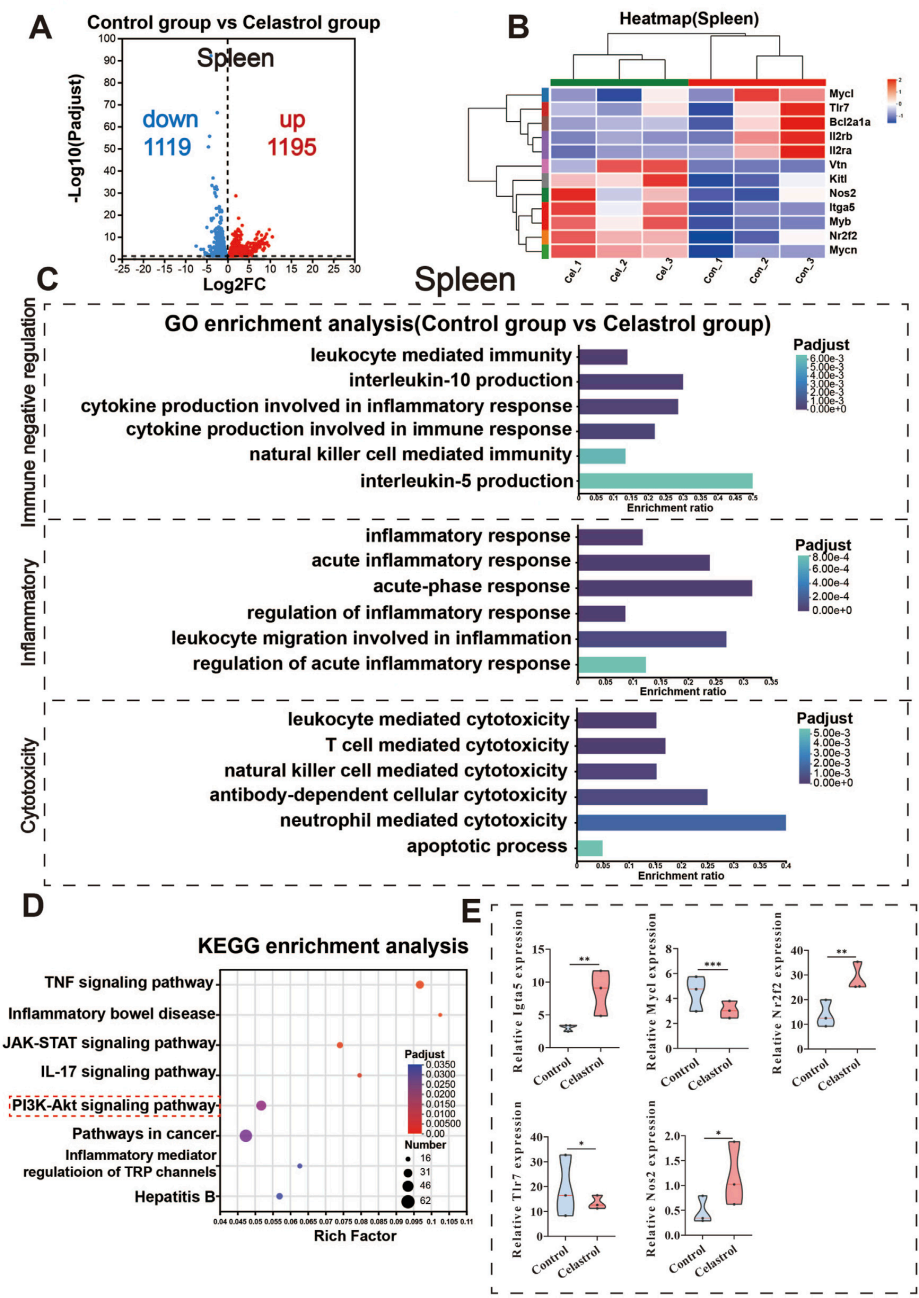
RNA-Seq analysis of the spleen between the celastrol and control group. **(A)** Volcanic map of differentially expressed genes in spleen; **(B)** Clustering heatmap of differentially expressed genes in spleen; **(C)** GO functional enrichment analysis of differentially expressed genes in spleen; **(D)** KEGG pathway enrichment analysis of differentially expressed genes in spleen; **(E)** Differential expression of key genes in the PI3K-Akt signaling pathway in spleen. (mean ± SD, n = 3, ^*^ *P* < 0.05, ^**^ *P* < 0.01, ^***^ *P* < 0.001).

Differential gene expression analysis was conducted using DESeq2, with the BH method for multiple testing correction. The criteria for identifying DEGs were |log2FC| ≥ 1 and padjust <0.05. Compared with the control group, 1,195 genes were upregulated in the spleen tissue of the celastrol group, including integrin alpha-5 (Itga5), while 1,119 genes were downregulated, including Tlr7. These results are presented in Figures 4A,B.

GO functional enrichment analysis results are presented in Figure 4C. Differentially expressed mRNAs in the spleen of the celastrol group were significantly enriched in 41 cellular components, 128 molecular functions, and 1,033 biological processes. Based on immune responses and systemic processes, the differentially expressed genes were categorized into three main groups: immune negative regulation, inflammation, and cell toxicity. Immune negative regulation included cytokine production and leukocyte-mediated immunity, both of which are negatively regulated. Inflammation involves processes like acute inflammatory response and leukocyte migration during the inflammatory response. Cell toxicity included positive regulatory processes mediated by various immune cells such as neutrophils and leukocytes.

The results of the KEGG pathway enrichment analysis are displayed in Figure 4D. The differentially expressed mRNAs in the spleen of the celastrol group were significantly enriched in 87 pathways (*P* < 0.05), including the PI3K-Akt signaling pathway, TNF signaling pathway, and IL-17 signaling pathway. Gene Set Enrichment Analysis (GSEA) revealed significant enrichment of the PI3K-Akt signaling pathway in the spleen with an FDR <0.05. Additional GSEA results for other signaling pathways are provided in the Supplementary Material (Supplementary Figures S8E, F).

Figure 4E shows the significantly enriched differential genes in the PI3K-Akt signaling pathway, such as upregulated genes Igta5, Nr2f2, and Nos2, and downregulated genes Mycl and Tlr7. The upregulation of Nos2 is closely associated with inflammation, potentially modulating immune cell function via nitric oxide (NO) signaling and enhancing the role of the PI3K-Akt signaling pathway in immune responses (Cinelli et al., 2020). The downregulation of Mycl is related to the inhibition of cell proliferation and survival (Bretones et al., 2015), suggesting negative regulation of the PI3K-Akt signaling pathway (Granato et al., 2016), which could impact immune system function. The downregulation of Tlr7 indicates that the PI3K-Akt pathway may play a role in negatively regulating immune responses (Liu et al., 2017; Singh et al., 2024), particularly in immune tolerance and cell metabolism. These significant changes in key genes within the PI3K-Akt signaling pathway further validate the earlier network toxicology analysis, suggesting that this pathway plays a crucial role in celastrol-induced immunotoxicity. The differential gene expression of selected genes in the PI3K-Akt signaling pathway within the spleen is shown in Supplementary Figure S6.

### 3.6 High dose triptolide activates PI3K-AKT mediated immunotoxicity

#### 3.6.1 Immunohistochemistry represents the activation status of the PI3K-Akt signaling pathway

**FIGURE 5:**
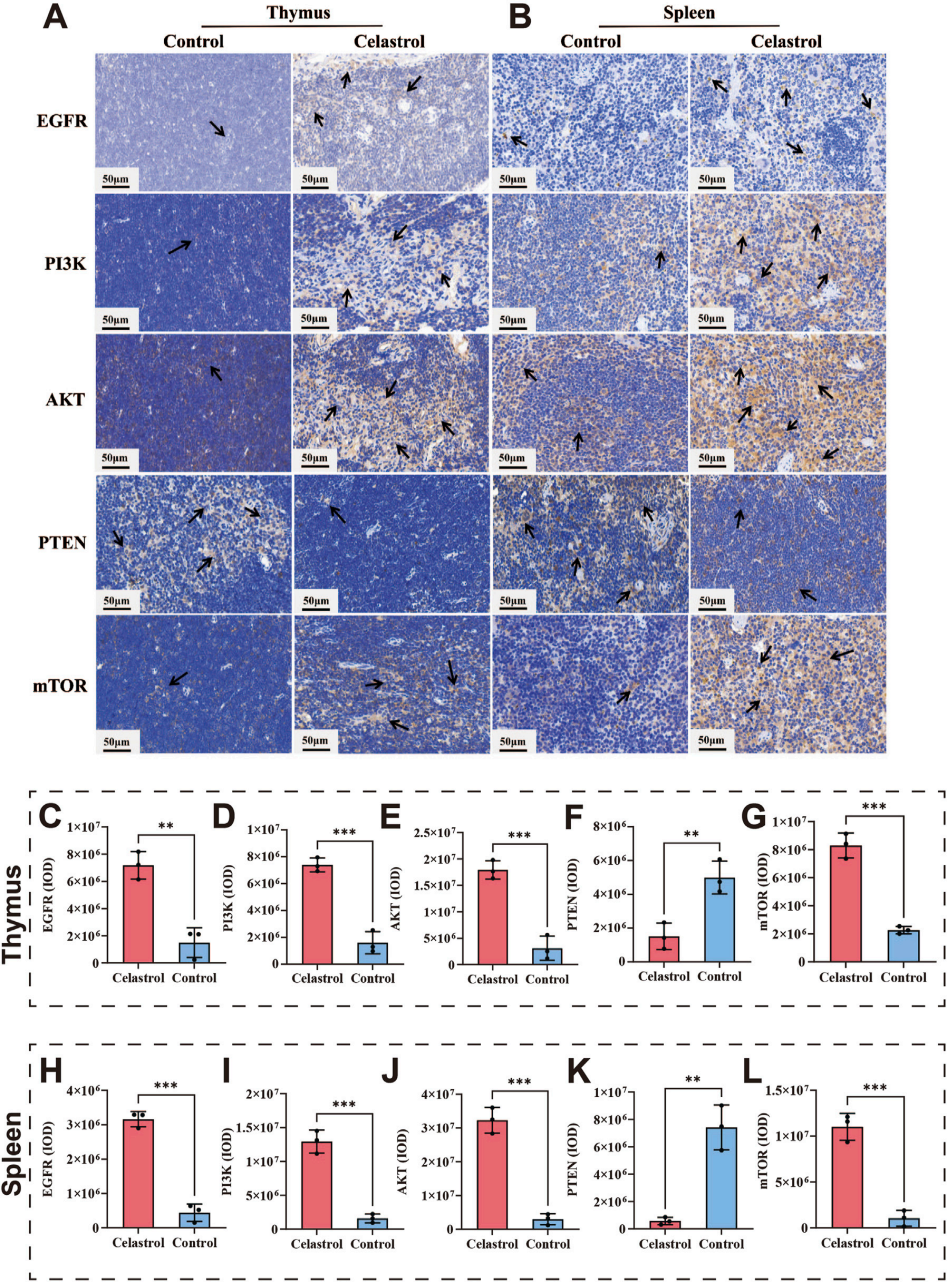
The PI3K-AKT pathway index revealed by IHC between celastrol and control group. **(A,B)** The IHC staining of thymus and spleen; **(C–G)**. The expression levels of core genes in the IHC analysis in the thymus; **(H–L)** The expression levels of core genes in the IHC analysis in the spleen (mean ± SD, n = 3, ^*^ *P* < 0.05, ^**^ *P* < 0.01, ^***^ *P* < 0.001).

Immunohistochemical techniques were employed to validate the expression of EGFR, PI3K, AKT, PTEN, and mTOR proteins in the thymus and spleen. The results are shown in Figure 5. The immunohistochemical findings for the thymus are presented in Figures 5C–F. Compared to the control group, the expression levels of PI3K, AKT, and mTOR were significantly increased in the celastrol group (*P* < 0.001). The expression of EGFR was also upregulated considerably (*P* < 0.01), while PTEN expression was significantly decreased (*P* < 0.01). The immunohistochemical findings for the spleen are presented in Figures 5G–J. Compared to the control group, the expression levels of EGFR, PI3K, AKT, and mTOR were significantly increased in the celastrol group (*P* < 0.001), and the expression level of PTEN was significantly decreased (*P* < 0.01). These findings suggest that celastrol disrupts immune system function by modulating signaling pathways involving the expression of EGFR, PI3K, AKT, PTEN, and mTOR proteins.

#### 3.6.2 The mIHC reveals the state of inflammatory factors and macrophage polarization

**FIGURE 6:**
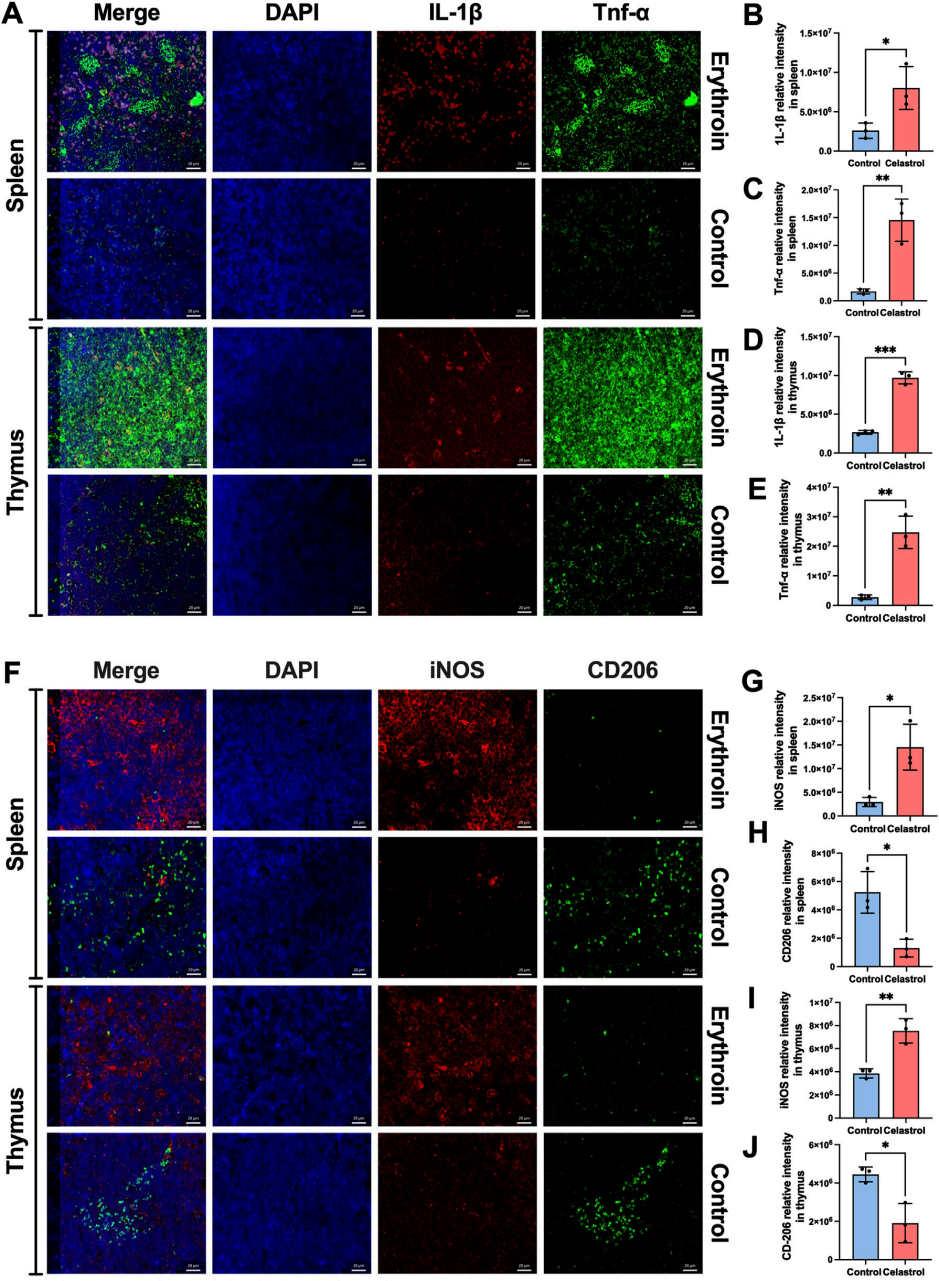
The inflammatory indexes revealed by mIHC between celastrol and control group. **(A)** The expression of IL-1β and Tnf-α in spleen and thymus; **(B–E)** The statistical analysis of relative expression of IL-1β and Tnf-α in spleen and thymus; **(F)**. The expression of iNOS and CD206 in spleen and thymus; **(G–J)** The statistical analysis of relative expression of iNOS and CD206 in spleen and thymus; (mean ± SD, n = 3, ^*^ *P* < 0.05, ^**^ *P* < 0.01, ^***^ *P* < 0.001).

To further investigate the toxic effects of high-dose celastrol on immunity and the immune system, we utilized mIHC technology to examine the inflammatory status of the spleen and thymus, as well as the polarization of macrophages. As shown in Figures 6A–E, spleen and thymus tissues from the celastrol group exhibited significantly higher expression levels of IL-1β and TNF-α compared the control group (*P* < 0.05). These results suggest that the underlying mechanism of toxicity induced by high-dose celastrol is associated with an excessively activated inflammatory state in these organs.

Additionally, the polarization of macrophages towards the pro-inflammatory M1 phenotype, as opposed to the anti-inflammatory M2 phenotype, reflects a state of immune hyperactivation. As shown in Figures 6F–J, the spleen exhibited a significantly higher number of M1 macrophages compared to M2 macrophages, and this number was higher than that observed in the control group (*P* < 0.05), indicating an immunotoxic state. Similarly, showed a higher ratio of M1 macrophages to M2 macrophages, with numbers exceeding those in the control group (*P* < 0.05).

#### 3.6.3 RT-qPCR analysis of celastrol activation of the PI3K-Akt signaling pathway mediating immunotoxicity

**FIGURE 7:**
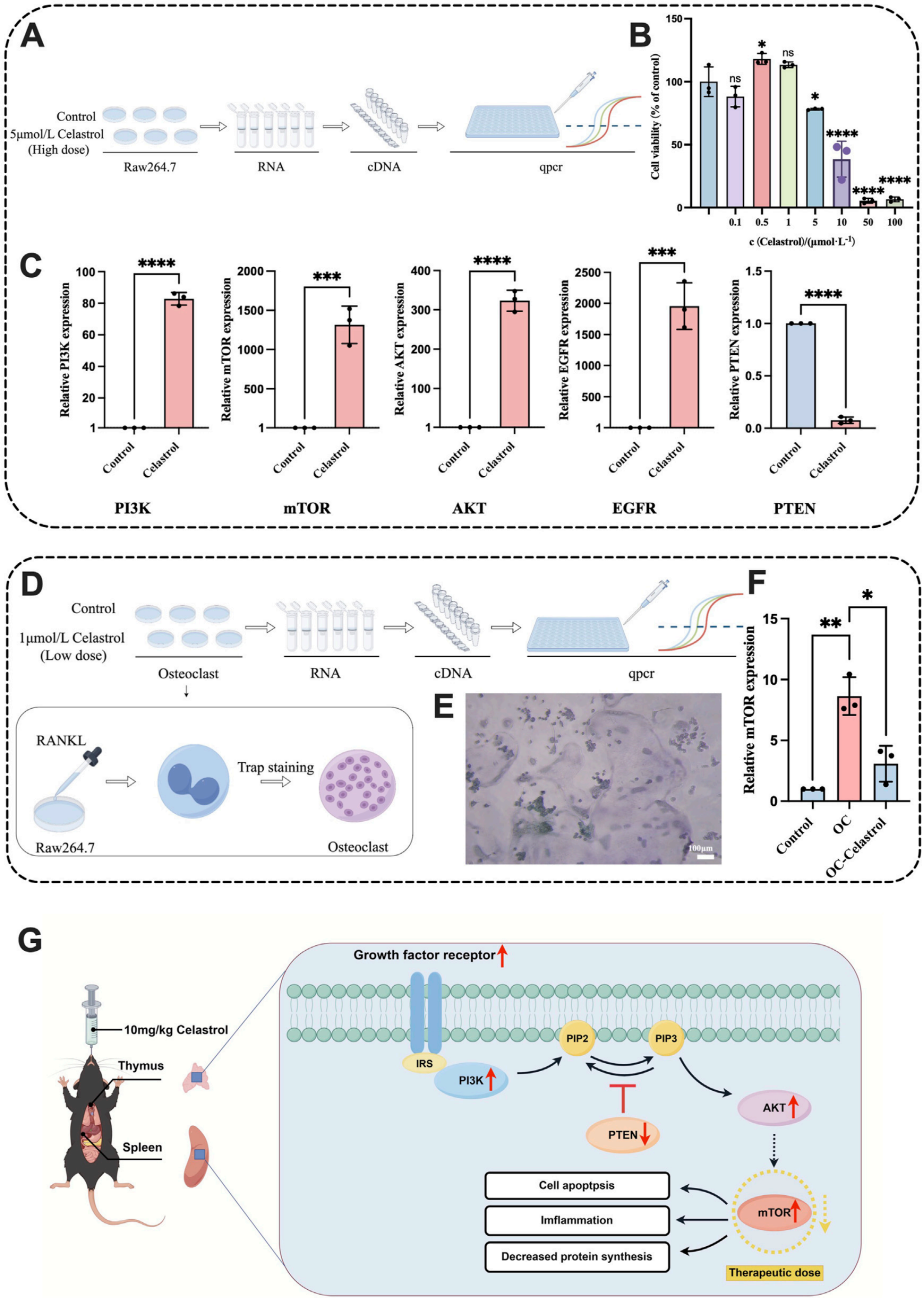
RT-qPCR analysis of Raw264.7 and osteoclasts treated with celastrol. **(A)** Schematic diagram of the RT-qPCR process of Raw264.7 with high dose celastrol. **(B)** CCK-8 assay results of Raw264.7 cells treated with celastrol (0, 0.1, 0.5, 1, 5, 10, 100 μmol/L) for 24h. **(C)** The expression levels of PI3K, mTOR, AKT, EGFR, and PTEN in the Raw264.7 RT-qPCR. **(D)** Schematic diagram of the RT-qPCR process of osteoclasts. **(E)** Image of positive Trap staining result for osteoclast. **(F)** The expression levels of mTOR in the osteoclast RT-qPCR. **(G)** PI3K-Aktsignaling pathway regulation diagram. (mean ± SD, n = 3, ^*^ *P* < 0.05, ^**^ *P* < 0.01, ^***^ *P* < 0.001, ^****^ *P* < 0.0001).

To explore the mechanism by which celastrol regulates the PI3K-Akt pathway to exert immunotoxicity, Raw264.7 macrophages were treated with 5 μmol/L high-dose celastrol, and a normal Raw264.7 control group was set up for qPCR experiments. The expression levels of five core proteins were analyzed to clarify the regulatory mechanism of the PI3K-Akt pathway. The experimental flowchart is shown in Figure 7A. The 5 μmol/L celastrol dose was selected based on the CCK-8 assay, which revealed that the survival rate of macrophages decreased at this concentration, indicating a certain level of cytotoxicity. No toxicity was observed in Raw264.7 cells at the 1 μmol/L celastrol concentration. The CCK-8 assay results are shown in Figure 7B. High-dose PCR results revealed that the expression levels of PI3K and AKT were significantly increased (*P* < 0.0001), while the expression of mTOR and EGFR was also significantly upregulated (*P* < 0.001). As a key protein inhibiting the PI3K-Akt pathway, PTEN expression was significantly decreased (*P* < 0.0001), confirming the activation of the PI3K-Akt pathway. The results are shown in Figure 7C.

### 3.7 Low dose of celastrol inhibits PI3K-Akt signaling pathway in osteoclast model

Osteoclasts are implicated in diseases such as osteoporosis, osteolysis, and rheumatoid arthritis (Veis and O'Brien, 2023; Wang et al., 2020), while celastrol has been shown to inhibit osteoclast formation and treat rheumatoid arthritis (Xu et al., 2021). To investigate the regulatory effect of celastrol on the PI3K-Akt signaling pathway in an osteoclast model, macrophages were induced to differentiate into osteoclasts using RANKL solution. Based on the results of the CCK-8 assay, a 1 μmol/L celastrol solution was selected to treat osteoclasts, and the regulatory effect of low-dose celastrol on the PI3K-Akt pathway in the osteoclast model was studied. The experimental flowchart is shown in Figure 7D. The well-differentiated osteoclasts were stained with TRAP, resulting in purple-red cytoplasm and blue nuclei, as shown in Figure 7E mTOR is a key regulatory target of celastrol-induced immunotoxicity. High-dose celastrol activates the PI3K-Akt-mTOR signaling pathway, leading to excessive activation of mTORC1, which in turn promotes pro-inflammatory T cell differentiation and M1 macrophage polarization, exacerbating inflammatory damage in the thymus and spleen (Byles et al., 2013; Delgoffe et al., 2009). At the same time, mTORC2 enhances its activity by phosphorylating Akt, forming a positive feedback loop to further amplify the inflammatory signal (Sarbassov et al., 2005). This abnormal activation leads to imbalance of immune cell apoptosis inhibition and increased oxidative stress, which eventually leads to lymphocyte exhaustion and histopathological damage (Perl, 2015). In contrast, low-dose celastrol inhibited mTOR activity, which may reduce immunotoxicity by restoring regulatory T cell function and inhibiting excessive activation of M1 macrophages (Zeng and Chi, 2017). The dose-dependent regulation mechanism of mTOR provides a molecular basis for explaining the bidirectional immune effect of celastrol and highlights its importance in toxicity threshold optimization (Efeyan and Sabatini, 2010), so we choose mTOR as the research object. PCR results showed that mTOR expression in osteoclasts was significantly upregulated compared to normal macrophages (*P* < 0.01), confirming successful osteoclast differentiation. The expression of mTOR in osteoclasts treated with low-dose celastrol was significantly lower than that in untreated osteoclasts (*P* < 0.05), as shown in Figure 7F. Therefore, low-dose celastrol inhibited the PI3K-Akt signaling pathway. Reducing mTOR expression and blocking mTOR signaling in osteoclasts can inhibit differentiation and bone resorption (Xu et al., 2021). These findings suggest that low-dose celastrol can achieve therapeutic effects by downregulating mTOR, inhibiting the PI3K-Akt pathway, and preventing excessive inflammation, showing an opposite regulatory effect compared to high-dose celastrol. Thus, low-dose celastrol is effective in treating rheumatoid arthritis.

## 4 Discussion

In this study, we integrated network toxicology, molecular docking, and biological methods to investigate celastrol’s immunotoxicity. Network toxicology analysis predicted the PI3K-Akt signaling pathway’s critical role. High-dose celastrol in mice caused thymus and spleen damage, and the results of RNA-Seq and immunohistochemistry confirmed that the PI3k-Akt pathway was over-activated. High-dose celastrol dysregulates the PI3K-Akt pathway, leading to immune system dysfunction. What’s more, mIHC revealed the excessive inflammation and macrophage activation status of the thymus and spleen.

It has been widely reported that high doses of celastrol induce toxicity in various organs (Song et al., 2023). Jiang et al. (Jiang et al., 2023) demonstrated significant hepatotoxicity and gastrointestinal toxicity in mice following high-dose celastrol administration. Liu et al. (Liu et al., 2019) explored the molecular mechanisms underlying celastrol-induced cardiac toxicity, which involves the induction of cardiomyocyte apoptosis at high doses. Wen et al. (Wen et al., 2023) showed that high-dose celastrol interferes with p21 transcriptional activity, leading to reproductive toxicity. Although these studies provide valuable insights into the toxic effects of celastrol, our study introduces a new dimension that emphasizes the dose-dependent immunomodulatory effects of celastrol. It was emphasized that the immunotoxicity of celastrol was dose-dependent. Specifically, we found that high-dose celastrol induces significant immunotoxicity in mice, causing substantial damage to the thymus and spleen, whereas low-dose celastrol may offer therapeutic benefits by modulating immune responses without causing significant toxicity. Furthermore, we investigated the expression changes of key genes in the PI3K-Akt signaling pathway under different doses of celastrol, revealing its potential role in regulating immune system function.

The overactivation of the PI3K-Akt pathway has been closely associated with the development of various autoimmune diseases, such as autoimmune hepatitis (Wang et al., 2023b), psoriasis (Patel and Mohan, 2005), and rheumatoid arthritis (Malemud, 2015; Noguchi et al., 2008). This dysregulation is believed to contribute to immune dysfunction through the induction of immune cell apoptosis and necroptosis (Choi et al., 2022; Hong et al., 2024). The PI3K-Akt pathway plays a crucial role in immune system regulation, and our study demonstrates that high-dose celastrol upregulates PIK3, AKT, mTOR, and EGFR, while downregulating PTEN, leading to abnormal activation of the PI3K-Akt pathway. This abnormal activation is likely to contribute to immune system imbalance by suppressing immune responses, reducing immune cell activity, and inducing inflammation. Interestingly, we observed that low-dose celastrol has the opposite effect on the PI3K-Akt pathway. It downregulates the expression of PIK3, AKT, mTOR and EGFR while up-regulating PTEN, thereby inhibiting pathway activation. This indicates that low-dose celastrol has the potential to regulate immune disorders, as shown in Figure 7G. This dose-dependent modulation of the PI3K-Akt pathway represents a significant finding, suggesting that low-dose celastrol may offer a promising therapeutic strategy to modulate immune responses without inducing toxicity.

In conclusion, this study provides novel insights into the dose-dependent effects of celastrol, particularly its dual action on the PI3K-Akt signaling pathway. At high doses, celastrol activates this pathway, while at low doses, it exerts inhibitory effects. These findings open new perspectives on the therapeutic potential of low-dose celastrol, suggesting that it may offer a safer and more effective approach to modulating immune responses. Such an approach could have significant implications for the clinical treatment of autoimmune and immune-related diseases. Future investigations should prioritize elucidating the molecular mechanisms governing celastrol’s dose-dependent efficacy differentials, particularly in defining the therapeutic window wherein PI3K-Akt pathway modulation elicits bidirectional regulatory outcomes. Concurrent exploration of potential tissue/organ specificity in these pharmacodynamic responses is imperative. Furthermore, systematic characterization of immunotoxicity detoxification pathways coupled with robust preclinical validation of its therapeutic index will be critical for advancing translational applications.

## 5 Conclusion

In this study, we employed an integrative approach combining network toxicology, molecular docking, and experimental biology to investigate celastrol’s immunotoxicity. Our findings revealed that high-dose celastrol ttriggers the PI3K-Akt signaling pathway, leading to thymus and spleen structural damage and immune system dysfunction in mice. Transcriptomic profiling and immunohistochemical analyses further confirmed the upregulation of critical pathway components in this pathway. Conversely, low-dose celastrol exhibited inhibitory effects on the PI3K-Akt signaling pathway, suggesting its potential therapeutic benefits in modulating immune responses without significant toxicity. These dose-dependent effects of celastrol offer new perspectives on its therapeutic optimization.

Our research contributes to the understanding of celastrol’s complex immunomodulatory actions, particularly its dual action on the PI3K-Akt signaling pathway. The overactivation of this pathway at high doses is likely to contribute to immune system imbalance, while its inhibition at low doses suggests a safer therapeutic strategy. Given the pivotal involvement of the PI3K-Akt signaling pathway in immune regulation and its association with autoimmune diseases, our findings have significant implications for the clinical treatment of these conditions. Future studies should focus on elucidating the underlying molecular mechanisms driving the differential effects of celastrol at varying doses and exploring its therapeutic efficacy and safety profile in translational models.

## Data Availability

The datasets presented in this study can be found in online repositories. The names of the repository/repositories and accession number(s) can be found blow: https://www.ncbi.nlm.nih.gov, Sequence Read Archive (SRA) submission: PRJNA1257543, https://www.ncbi.nlm.nih.gov/bioproject/PRJNA1257543.

## Abbreviations

ETCM, The Encyclopedia of Traditional Chinese Medicine; CTD, Comparative Toxicogenomics Database; OMIM, Online Mendelian Inheritance in Man; PPI, Protein-protein Interaction; DC, Degree Centrality; BC, Betweenness Centrality; GO, Gene Ontology; KEGG, Kyoto Encyclopedia of Genes and Genomes; BP, Biological Process; CC, Cellular Component; MF, Molecular Function; WBC, White Blood Cells; NEUT, Neutrophils; LY, Lymphocytes; ALT, Alanine Aminotransferase; ALP, Alkaline Phosphatase; TBIL, Total Bilirubin; BUN, Blood Urea Nitrogen; SCR, Serum Creatinine; HE, Hematoxylin-eosin; TRAP, Tartrate-resistant Acid Phosphatase; GSEA, Gene Set Enrichment Analysis; IHC, Immunohistochemical; mIHC, Multiplex Immunohistochemical; RT-qPCR, Real-time quantitative polymerase chain reaction.
