# Supplementary material for: Dose-dependent immunotoxic mechanisms of celastrol via modulation of the PI3K-Akt signaling pathway
Source: Front Pharmacol. 2025 May 27;16:1567193. doi: 10.3389/fphar.2025.1567193 (PMC12149175; doi:10.3389/fphar.2025.1567193)
Supplement: Supplementary file 1 [file DataSheet1.docx]

**Supporting Information**

**Dose-dependent immunotoxic mechanisms of celastrol via modulation of the PI3K-Akt signaling pathway**

*Shaohui Geng^1,†^, Jingqi Wen^2,†^, Chunli Shen^3,†^, Li Liu^1,†^*, *Yijin Jiang^4^, Jingyuan Fu^4^, Yiwei Guan^4^, Zi Ye^4^, Yuanhao Wu^5,*^, Chen Li^6,*^, Guangrui Huang^1,*^*

^1^ School of Life Science, Beijing University of Chinese Medicine, Beijing 100029, China;

^2^ School of Traditional Chinese Medicine, Beijing University of Chinese Medicine, Beijing 100029, China;

^3^ School of Acupuncture, Moxibustion and Tuina, Beijing University of Chinese Medicine, Beijing 100029, China;

^4^ School of Chinese Pharmacy, Beijing University of Chinese Medicine, Beijing, China, Beijing 100029, China;

^5^ The First Affiliated Hospital of Tianjin University of Traditional Chinese Medicine, Tianjin, 300381, China;

^6^ Department of Dermatology, Tianjin Institute of Integrative Dermatology, Tianjin Academy of Traditional Chinese Medicine Affiliated Hospital, Tianjin 300120, China;

^†^These authors have contributed equally to this work and share first authorship

^*^Corresponding Authors: Guangrui Huang: hgr@bucm.edu.cn; Chen Li: [casio1981@163.com](mailto:casio1981@163.com); Yuanhao Wu: doctor.wuyh@gmail.com

**Sup Table 1** Important target information on the toxic effects of celastrol on thymus and spleen in PPI network

| Name | Degree | Betweenness Centrality | Closeness Centrality |
| --- | --- | --- | --- |
| EGFR | 58 | 0.0024 | 1.0000 |
| MTOR | 57 | 0.0022 | 0.9831 |
| PTEN | 57 | 0.0021 | 0.9831 |
| AKT1 | 58 | 0.0024 | 1.0000 |
| PIK3R1 | 57 | 0.0017 | 0.9667 |
| TGFB1 | 57 | 0.0022 | 0.9831 |
| TP53 | 58 | 0.0024 | 1.0000 |
| ALB | 57 | 0.0020 | 0.9831 |
| ANXA5 | 56 | 0.0017 | 0.9667 |
| CASP3 | 58 | 0.0024 | 1.0000 |
| FN1 | 56 | 0.0021 | 0.9667 |
| STAT1 | 56 | 0.0019 | 0.9667 |
| JUN | 58 | 0.0024 | 1.0000 |
| TNF | 57 | 0.0020 | 0.9831 |
| GAPDH | 58 | 0.0024 | 1.0000 |
| IL6 | 58 | 0.0024 | 1.0000 |
| ESR1 | 56 | 0.0019 | 0.9667 |
| BCL2 | 58 | 0.0024 | 1.0000 |
| SRC | 58 | 0.0024 | 1.0000 |
| BCL2L1 | 57 | 0.0020 | 0.9831 |
| MYC | 57 | 0.0022 | 0.9831 |
| CTNNB1 | 57 | 0.0022 | 0.9831 |
| PPARG | 56 | 0.0017 | 0.9667 |
| MMP9 | 56 | 0.0017 | 0.9667 |


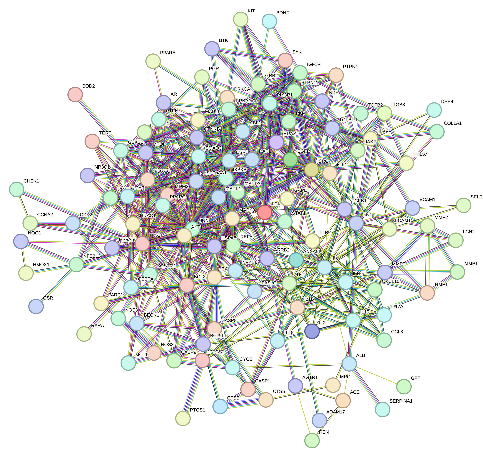


**Sup Figure 1.** PPI relationship of thymus and spleen toxicity induced by celastrol.

**
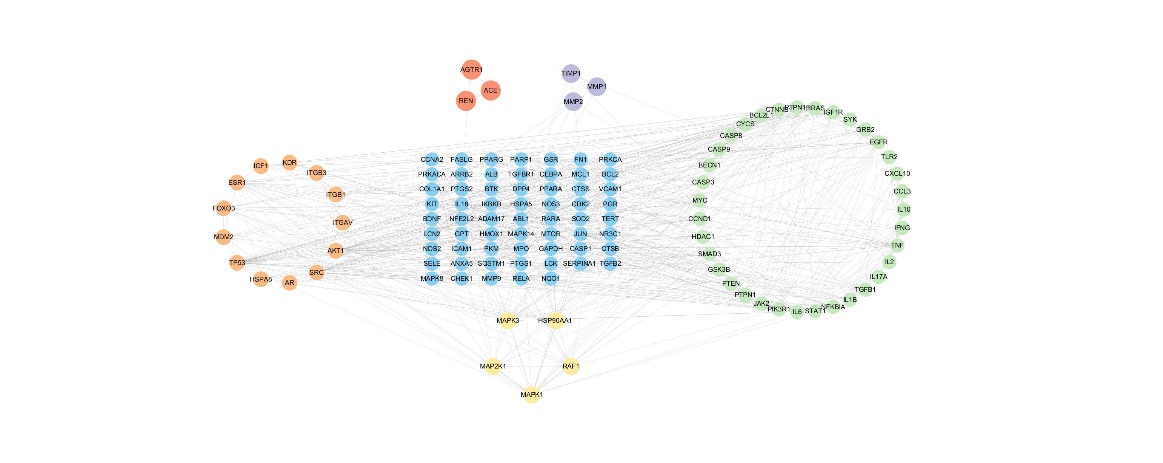
**

**Sup Figure 2. P**PI clustering of thymus and spleen toxicity induced by celastrol.


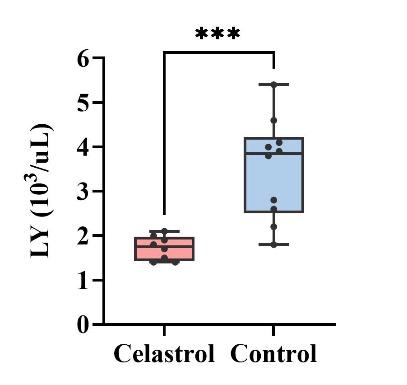


**Sup Figure 3.** The number of lymphocytes in the celastrol group and control group.


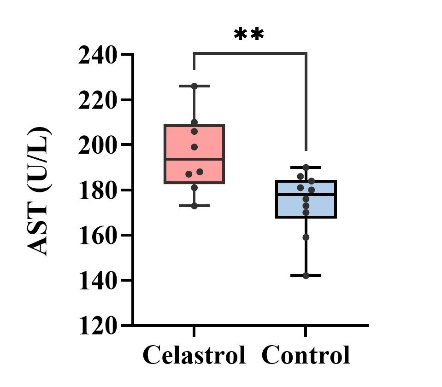


**Sup Figure 4.** AST content in the celastrol group and control group.


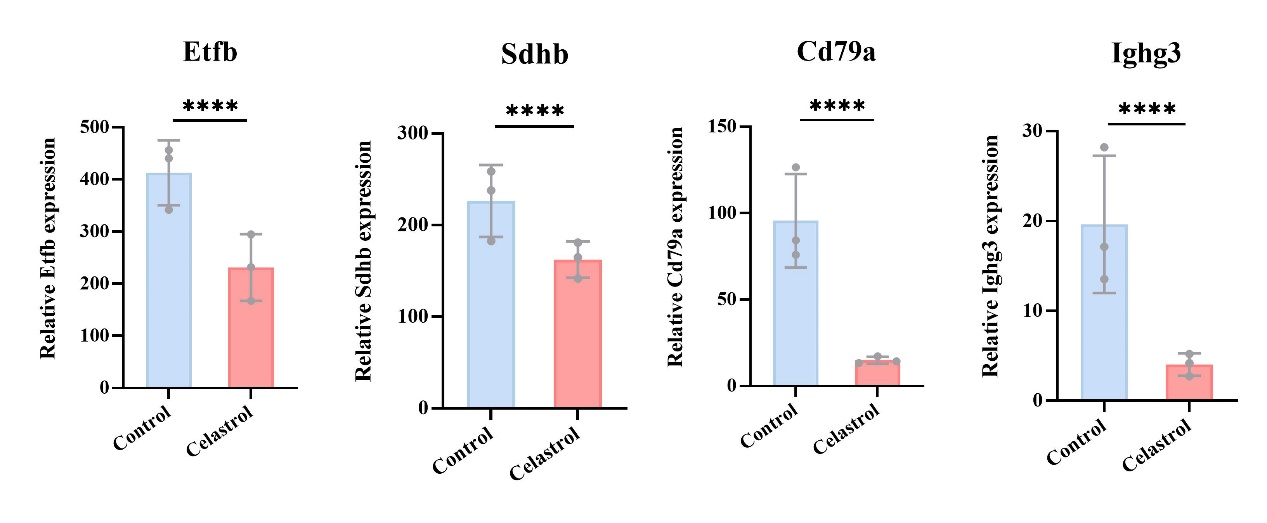


**Sup Figure 5.** Differential gene expression in the PI3K-Akt signaling pathway of thymus.


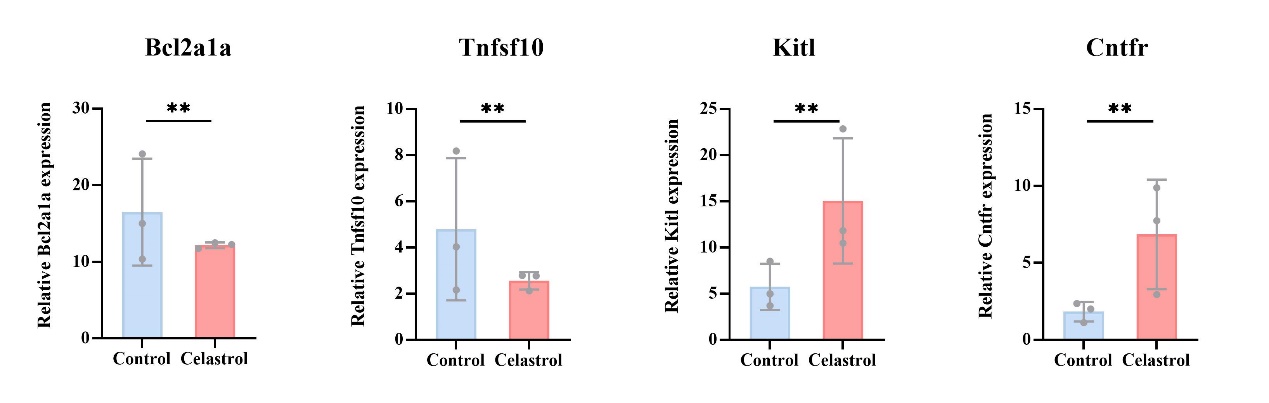


**Sup Figure 6.** Differential gene expression on the PI3K Akt signaling pathway in spleen

**
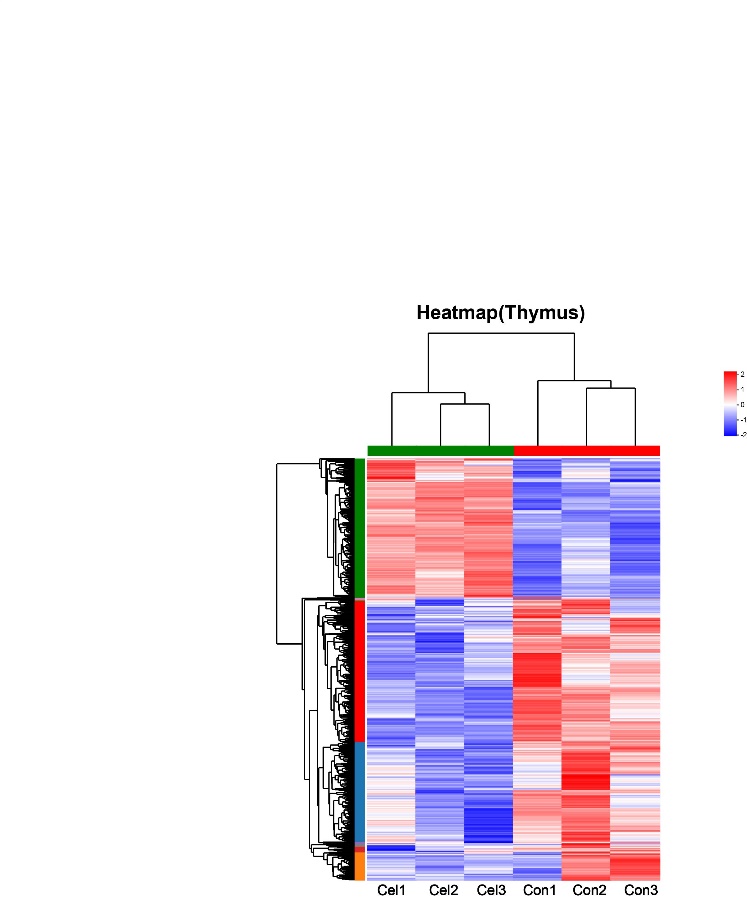

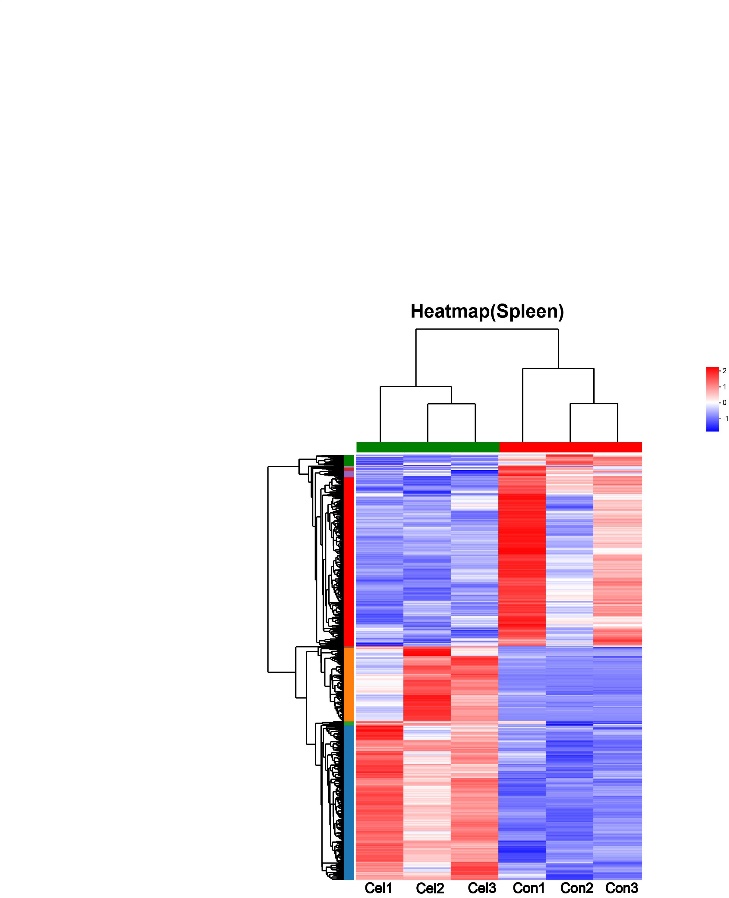
**

**Sup Figure 7.** Cluster heatmap of differentially expressed genes in thymus and spleen.

**
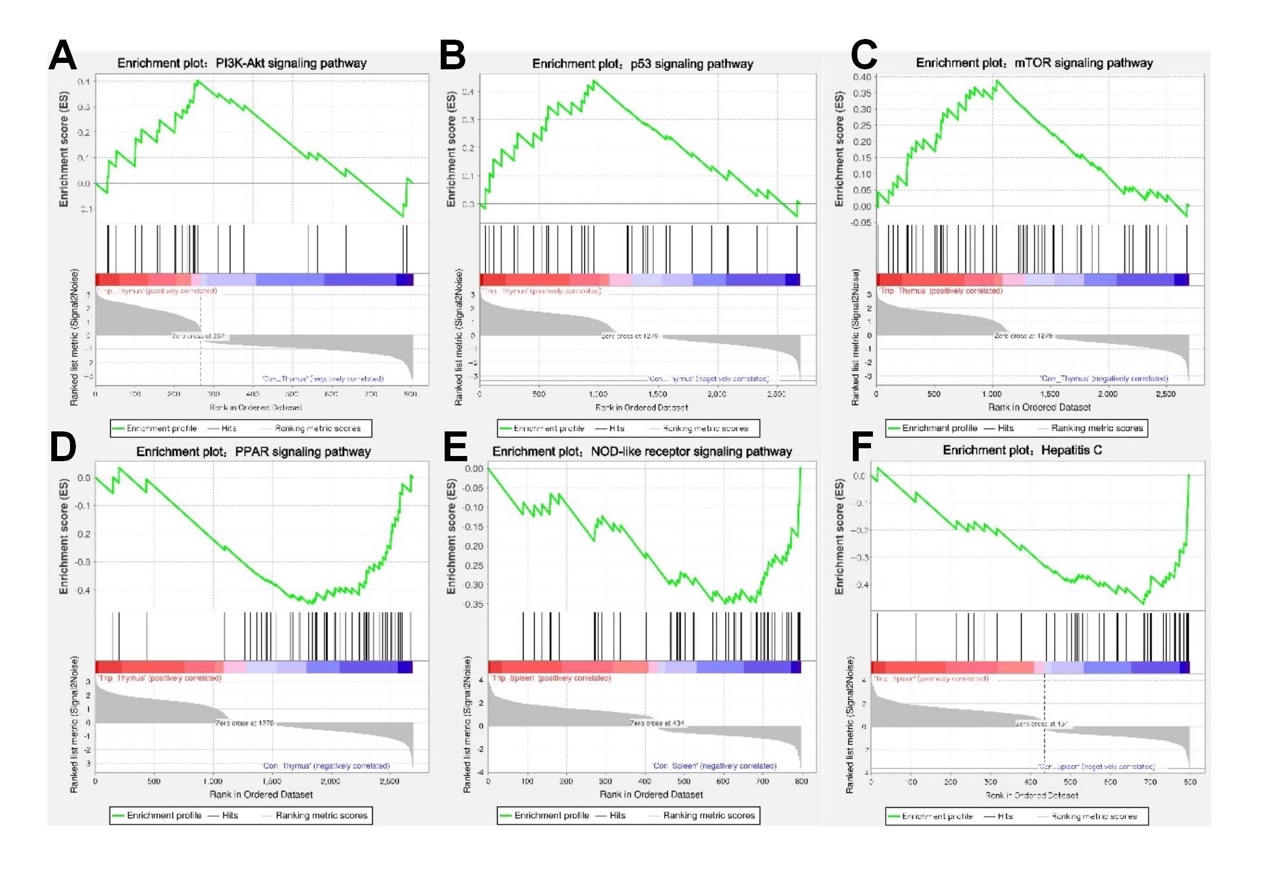
**

**Sup Figure 8.** GSEA analysis of significant differential signaling pathways.

A, B, C, D GSEA analysis of signaling pathways in spleen; E.F GSEA analysis of signaling pathways in thymus.


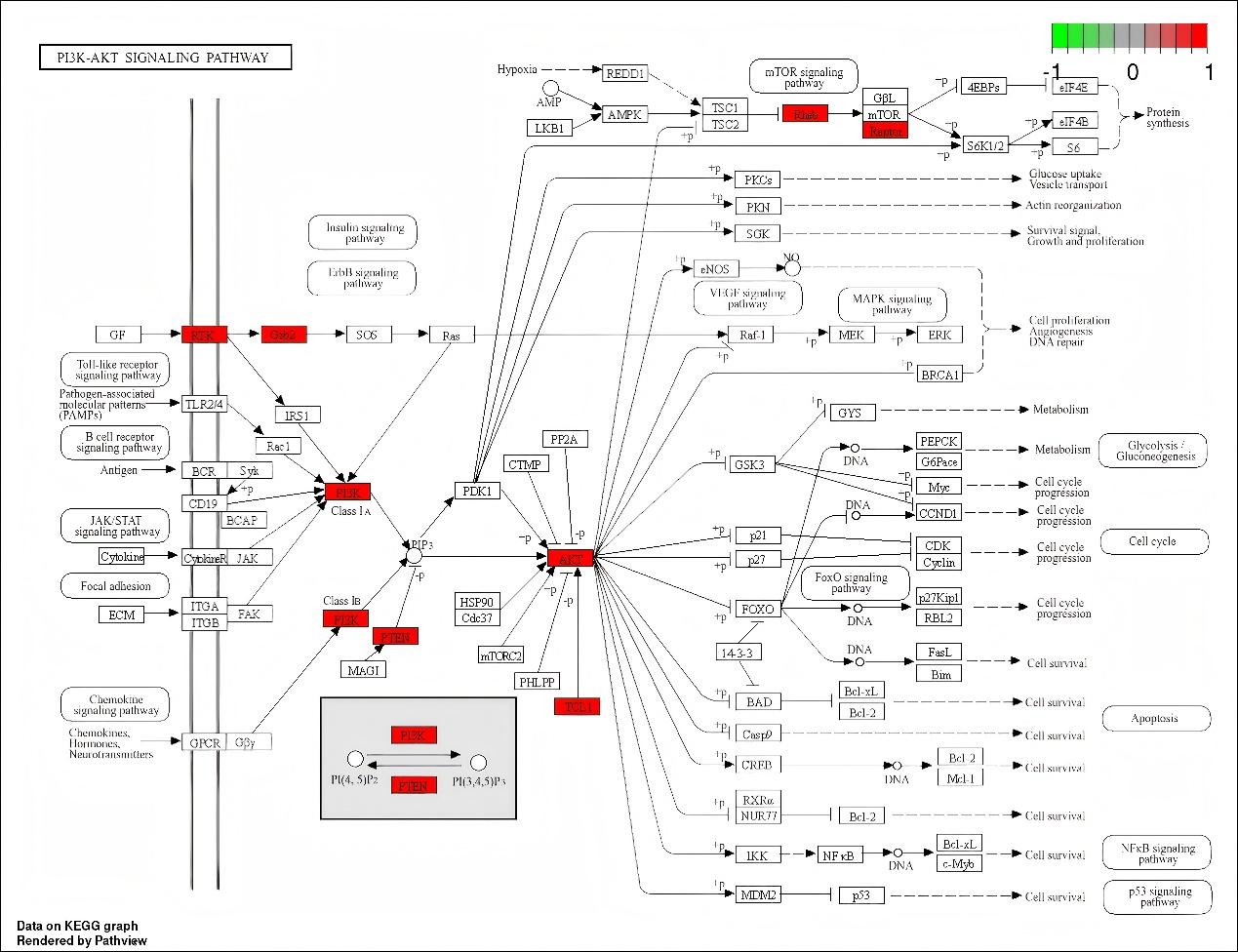


**Sup Figure 9.** The PI3K Akt signaling pathway map enriched by KEGG was depicted, illustrating the mechanism of immunotoxicity induced by celastrol.


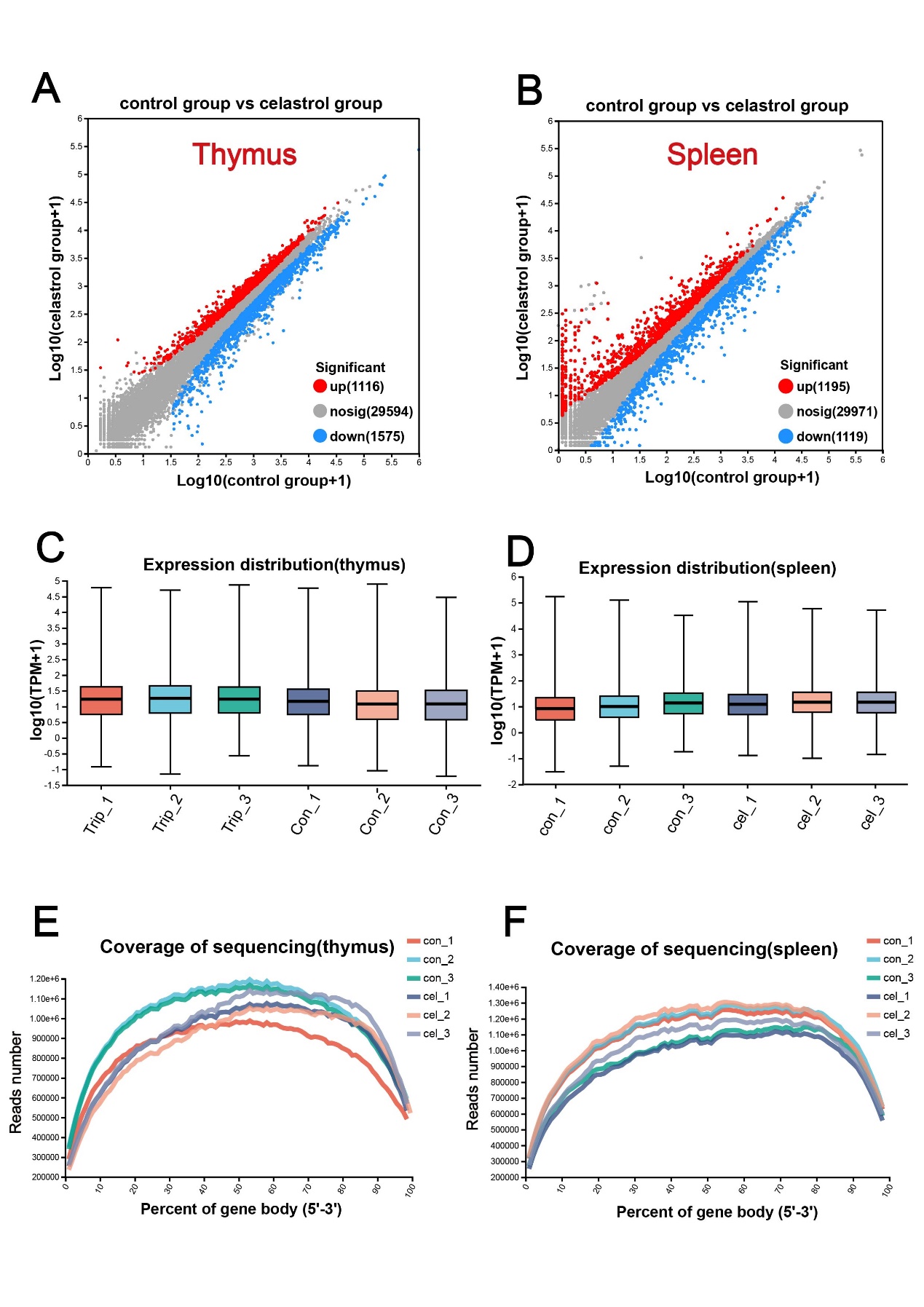


**Sup Figure 10.** Transcriptome sequencing quality control of thymus and spleen.

A, B. Volcanic map of differentially expressed genes in thymus and spleen; C, D. The expression levels of various transcripts in the thymus and spleen; E, F. Transcriptome sequencing coverage analysis of thymus and spleen.

**
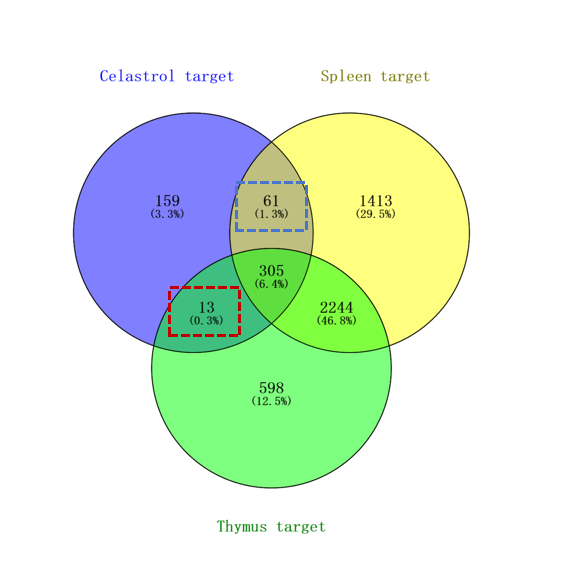
**

**Sup Figure 11.** The venn map of celastrol-spleen-thymus targets.

**Sup Table 2 C**elastrol-spleen-thymus specific targets

| Description | Number | Specific targets |
| --- | --- | --- |
| celastrol-spleen specific targets (blue box) | 61 | THRA, THRB, TBXAS1, CMA1, ATP12A, STS, CES2, CA2, APOA2, DHODH, SULT2A1, DUSP6, HDAC8, BCAT2, IMPDH2, NR1I3, CES1, CCNT1, GSTA1, FHIT, LTA4H, GM2A, ADH5, RARB, CBR1, KYAT1, FECH, GLO1, ALAD, SULT1E1, HSP90AB1, ACADM, GPI, FKBP1B, RNASE2, MAPK12, EIF4E, TPH1, GSTM2, HEXB, BAG3, CDA, CITED2, DNAJA1, GCLC, GCLM, HK2, HSPA1B, MDH2, OGDH, PDP1, PPIF, RPS6KB1, SLC3A2, TXNRD1, WNT3, WNT7B |
| celastrol-thymus specific targets (red box) | 13 | PTPRF, PTGDR2, CALCRL, CPB2, HSD11B1, CTNNA1, CTSF, NMNAT1, AXIN2, GTPBP2, LPIN1, TCF7, UGDH |
